# The Peach v2.0 release: high-resolution linkage mapping and deep resequencing improve chromosome-scale assembly and contiguity

**DOI:** 10.1186/s12864-017-3606-9

**Published:** 2017-03-11

**Authors:** Ignazio Verde, Jerry Jenkins, Luca Dondini, Sabrina Micali, Giulia Pagliarani, Elisa Vendramin, Roberta Paris, Valeria Aramini, Laura Gazza, Laura Rossini, Daniele Bassi, Michela Troggio, Shengqiang Shu, Jane Grimwood, Stefano Tartarini, Maria Teresa Dettori, Jeremy Schmutz

**Affiliations:** 1 Consiglio per la ricerca in agricoltura e l’analisi dell’economia agraria (CREA), Centro di Ricerca per la Frutticoltura, 00134 Rome, Italy; 2 HudsonAlpha Institute of Biotechnology, Huntsville, AL USA; 3 Department of Agricultural Sciences (DipSA), University of Bologna, Bologna, Italy; 4 Department of Agricultural and Environmental Sciences (DISAA), University of Milan, Milan, Italy; 5 Parco Tecnologico Padano, Via Einstein, 26900 Lodi, Italy; 6 Research and Innovation Centre, Fondazione Edmund Mach (FEM), 38010 San Michele all’Adige, TN Italy; 7 U.S. Department of Energy, Joint Genome Institute, Walnut Creek, CA 94598 USA; 8 Present address: Consiglio per la ricerca in agricoltura e l’analisi dell’economia agraria (CREA), Centre of Research for Industrial Crops, 40128 Bologna, Italy; 9 Present address: Consiglio per la ricerca in agricoltura e l’analisi dell’economia agraria (CREA), Research Unit for Cereal Quality, Rome, Italy

**Keywords:** *Prunus persica*, WGS assembly, SNPs, SSRs, Linkage mapping, NGS resequencing, Gap patching, Recombination rates, Centromeric regions

## Abstract

**Background:**

The availability of the peach genome sequence has fostered relevant research in peach and related *Prunus* species enabling the identification of genes underlying important horticultural traits as well as the development of advanced tools for genetic and genomic analyses. The first release of the peach genome (Peach v1.0) represented a high-quality WGS (Whole Genome Shotgun) chromosome-scale assembly with high contiguity (contig L50 214.2 kb), large portions of mapped sequences (96%) and high base accuracy (99.96%). The aim of this work was to improve the quality of the first assembly by increasing the portion of mapped and oriented sequences, correcting misassemblies and improving the contiguity and base accuracy using high-throughput linkage mapping and deep resequencing approaches.

**Results:**

Four linkage maps with 3,576 molecular markers were used to improve the portion of mapped and oriented sequences (from 96.0% and 85.6% of Peach v1.0 to 99.2% and 98.2% of v2.0, respectively) and enabled a more detailed identification of discernible misassemblies (10.4 Mb in total). The deep resequencing approach fixed 859 homozygous SNPs (Single Nucleotide Polymorphisms) and 1347 homozygous indels. Moreover, the assembled NGS contigs enabled the closing of 212 gaps with an improvement in the contig L50 of 19.2%.

**Conclusions:**

The improved high quality peach genome assembly (Peach v2.0) represents a valuable tool for the analysis of the genetic diversity, domestication, and as a vehicle for genetic improvement of peach and related *Prunus* species. Moreover, the important phylogenetic position of peach and the absence of recent whole genome duplication (WGD) events make peach a pivotal species for comparative genomics studies aiming at elucidating plant speciation and diversification processes.

**Electronic supplementary material:**

The online version of this article (doi:10.1186/s12864-017-3606-9) contains supplementary material, which is available to authorized users.

## Background

The WGS (Whole Genome Shotgun) approach for sequencing complex eukaryotic genomes [1, 2] has contributed to the assembling many genomes of non-model and crop species. Poplar [3] and grape [4] were the first plant genomes sequenced with this approach. The advantages of WGS sequencing, as compared to the BAC by BAC (BAC, Bacterial Artificial Chromosome) approach [5–7] are the speed of sequencing and the reduced cost. However, a weakness of the WGS sequencing approach is it tends to produce a more fragmented assembly with reduced contiguity, also coupled with the risk of large-scale misassemblies. This is especially true for complex eukaryotic genomes and the assembly process can be confounded with recent duplication events (either segmental or Whole Genome Duplication, WGD), large regions of expanded repeats (up to 85% of the genome in species such as corn and wheat [8, 9]), and residual heterozygosity. The result is the production of a fragmented sequence with poor contiguity metrics such as the N50 (number of DNA stretches that contain half of the genome) and the L50 (the shortest sequence length at 50% of the genome). The potential issues are greater if a highly heterozygous individual is chosen as reference [10, 11]. However, in the BAC by BAC approach, the use of local sequence information (i.e. the single BAC clone) mitigates the risk of large-scale misassembly. The advent of Next Generation Sequencing (NGS) technologies has exacerbated WGS assembly drawbacks, typically producing a more fragmented assembly. NGS produces shorter sequence reads compared to the Sanger method, making genome assembly more difficult and requiring the development of a range of dedicated bioinformatics tools and novel alignment algorithms [12]. A *de novo* short-read NGS assembly needs high genome coverage, mainly to overcome the reduced overlap length and improve the contiguity of the resulting assembly [13]. Henson et al. [14] calculated that by increasing the reads length from 50 bp to 1000 bp, the contig L50 value of the human genome can theoretically increase from 3 kb to about 9,000 kb. Moreover, the lack of a chromosome-scale assembly, combined with putative misassemblies (usually undetected in non-anchored WGS genomes) precludes extensive use in evolutionary and comparative genomics studies, as well as Genome Wide Association Studies (GWAS). The availability of a chromosome-scale assembly is, therefore, crucial to maximally leverage the advantages of the WGS sequences. The coupling of WGS genome assembly with highly saturated and high resolution molecular genetic maps has been proposed to cope with the lack of chromosome-scale WGS genomes [15–17]. Genetic maps enable the reconstruction of a chromosome-scale sequence by positioning WGS scaffolds in their correct order and orientation to arrange them in long stretches of DNA, representing the individual chromosomes, called “pseudomolecules” or “pseudochromosomes”. Inconsistencies between the position of markers on the map and in the assembly can highlight putative misassembled sequences that can be further resolved by breaking the chimeric scaffolds and rearranging the broken pieces in their correct order and orientation. Fragmented genome assemblies, such as those obtained with short reads NGS data, need dense genetic maps for a large fraction of the assembled sequence to be anchored on chromosomes.

The availability of WGS genome assemblies in many species combined with NGS platforms has fueled variant discovery through alignment of resequenced reads of different accessions to the reference genome. Millions of Single Nucleotide Polymorphisms (SNPs) and small insertions/deletions (indels) dispersed throughout the genome have been discovered in different species [18–20]. This discovery, coupled with high-throughput genotyping technologies, such as SNP arrays [21–26] and genotyping by sequencing (GBS) [27–30], has accelerated the construction of high-resolution genetic maps, enabling map-sequence integration of WGS scaffolds in highly fragmented *de novo* NGS assemblies. Medium and high-throughput genotyping tools have been developed in *Prunus* for peach [31] and cherry [32] and in other Rosaceae species such as apple [21, 33] and strawberry [22].

To overcome the limitations of short read sequencing technologies (such as Illumina) third generation methodologies, based on single molecule sequencing, have been recently released such as the one of Pacific Bioscience [34] and Moleculo [35]. These methodologies, are capable of obtaining much longer reads, up to 50 kb [36], with the trade-off of an increase in error rates of 13-15% [37, 38] in comparison to 0.2–0.8% in Illumina short reads [38]. To solve this problem, several strategies have been proposed such as the integration of long reads with more accurate NGS reads. This hybrid sequencing strategy has been recently used in pineapple [39, 40] apple [41] and *Arabidopsis thaliana* L*er* [42].

The peach [*Prunus persica* (L.) Batsch] genome sequence was obtained by the International Peach Genome Initiative (IPGI [20]) and is an 8.5-fold WGS high quality draft sequence [43] with long contiguity, high base accuracy, and a large portion of sequences mapped on chromosomes. Sequences were obtained using the Sanger methodology and a complete homozygous reference accession, the ‘Lovell’ double haploid PLov2-2n. The *Prunus* reference map (TxE [44–46]) was used to anchor the first release of the peach genome (Peach v1.0) obtaining eight pseudomolecules representing the eight *Prunus* chromosomes. Approximately half of the markers were placed by genotyping only six seedlings using the BIN mapping strategy [47, 48]. Thus, even if the BIN mapped markers were useful to assign scaffolds to chromosomes and check scaffold integrity, in most cases they did not provide sufficient information for anchoring the scaffolds on chromosomes. Moreover, the TxE map is an interspecific map and was obtained with a limited number of individuals (88) resulting in a reduced recombination frequency in some regions [49, 50], providing only a rough estimation of the recombination frequency at a short physical distance. The anchoring markers (i.e. those having sequence information associated) are unevenly distributed along the linkage groups leaving portions of the genome uncovered. Later analyses of the peach genome revealed that 4% of the sequence of the Peach v1.0 (with ten major scaffolds larger than 300 kb) was not included in the pseudomolecules; with ten scaffolds (7% of the total sequence) anchored with unknown orientation and a large number of mapped scaffolds had no markers on their ends (>500 kb), making it difficult to detect putative chimerism [20].

In this work, we describe the efforts aimed at improving the peach chromosome-scale build (Peach v1.0 [20]) using a set of linkage maps and resequencing the reference accession (‘Lovell’ double haploid). Two mapping strategies were used: i) a targeted approach where markers (Simple Sequence Repeats, SSRs, and SNPs) were targeted in specific regions of the peach genome (i.e. in map gaps, unmapped, not oriented scaffolds, and uncovered scaffold ends) and mapped in two already available linkage maps [45, 51]; ii) a whole genome approach that made use of the IPSC 9 K SNP array v1 [31] to genotype a large set of progeny [50, 52, 53]. Moreover, Illumina NGS resequencing of the reference accession at high coverage was performed to correct sequencing errors (false SNPs and indels) and to close a number of gaps in the Peach v1.0 assembly increasing the contiguity of the final peach genome.

## Methods

### Plant material, DNA extraction and quality test

Four biparental mapping populations were used to refine the peach genome (Peach v1.0):

i) 67 seedlings of the ‘Texas’ x ‘Earligold’ F_2_ population (TxE [46]), an interspecific cross between almond and peach, maintained at the experimental station of CREA-FRU in Rome, Italy (latitude: from 41°47'43.72"N to 41°47'46.75"N; longitude: from 12°33'48.78"E to12°33'52.58"E);
ii) 242 seedlings of the peach selection IF7310828 x Ferganensis BC_1_ population (PxF [52]) maintained at the experimental station of CREA-FRU;
iii) 305 seedlings of the ‘Contender’ x ‘Ambra’ F_2_ population (CxA [51]) maintained in a farm belonging to the Municipality of Castel San Pietro (Bologna, Emilia Romagna, Italy) leased to ASTRA (latitude: from 44u24944.180 N to: 44u24930.080 N; longitude: from 11u35947.210E, to: 11u3692.000E);
iv) 62 seedlings of the Maria Dolce x SD81 F_1_ cross (MDxSD) maintained at the experimental station of CREA-FRU.

Young leaves were collected from each seedling and lyophilized. DNA was extracted with the DNeasy Plant Mini Kit (QIAGEN), quantified with the NanoDrop spectrophotometer (Thermo Fisher Scientific, Waltham, MA, USA) and with the PicoGreen® Assay (Thermo Fisher Scientific) for samples genotyped on the IPSC 9 K SNP array. For Sequenom analysis, DNA was extracted from the seedlings of the CxA progeny after Mercado et al. [54].

### Target SSRs identification and mapping in TxE progeny

Target SSRs were selected from among the 63,145 identified within the Peach v1.0 genome sequence [55] to increase marker density in order to leverage in large unanchored scaffolds, and correct order/orientation of anchored scaffolds. Sequences of ~600 bp flanking both sides of the repeated motif were first blasted against Peach v1.0, using the BLAST facility available on Phytozome [56, 57], and only non-repetitive regions were selected to design specific primer pairs by the Primer 3 software [58]. Only single locus SSR markers were used for further analysis. The selected SSR primers were first tested on the BIN set and parents of the TxE progeny. Only the most physically distant polymorphic markers in each target region were genotyped on the whole TxE progeny. To check for scaffold integrity, some SSRs were developed in the distal region of uncovered scaffold ends. In the presence of suspected chimeric regions, further markers spaced approximately every 100 kb were identified, developed, and mapped to restrict the size of misassembly containing regions. Primer sequences and features are listed in Additional file 1: Table S1.

All PCR reactions were carried out in a 10 μL volume with a final concentration of 1x PCR buffer, 1.5 mM MgCl_2_, 200 μM of each dNTP and 0.1 μM of each primer, 10 ng genomic DNA, and 0.5U of Platinum®Taq DNA Polymerase (Invitrogen TermoFisher). The amplification profile was: one cycle at 94 °C for 5 min, followed by 10 touchdown cycles, with a decrement of the annealing step temperature of 0.5 °C/cycle, starting with a denaturation at 94 °C for 30 s, an annealing step five degrees above the primer-specific annealing temperature (Ta °C, reported in Additional file 1: Table S1) for 30 s, and an elongation at 72 °C for 30 s, followed by 25 cycles at 94 °C for 30 s, Ta °C for 30 s, 72 °C for 30 s, and a final elongation of 30 min at 72 °C. PCR products were then separated on a 3% high-resolution agarose gel (MetaPhor™Agarose, Lonza) in TBE 1x with a voltage of ∼ 5 V/cm and stained with the GelRed™ (Biotium). Markers that could not be easily scored on agarose gel were separated by capillary electrophoresis on a CEQ 8000 Genetic Analyzer (Beckmann Coulter).

SSRs genotyped in the whole TxE progeny were integrated with the previous TxE dataset [45] and mapped using the software MAPMAKER [59], grouping them at a LOD score higher than 5. They were located by using the TRY and RIPPLE commands. After mapping, the ERROR DETECTION command of MAPMAKER was used and putative double recombinants were manually checked.

### SNP identification and mapping in the CxA progeny

The F_1_ parent of the CxA progeny was resequenced with an Illumina platform (Project SRA0532230, Accession # SRX150230 [20]). The CLC Genomics Workbench 5.5 (CLC Bio, Aarhus, Denmark) was used for read alignment and SNP calling. Only reads aligning to a single location with at least 92% identity over at least 90% of their length were considered. For SNP calling, variants were retained when: i) the coverage ranged between 0.5 and 2 X of the average coverage (computed excluding zero coverage regions); ii) minor allele frequency > 30%; iii) the polymorphic nucleotide Phred-scaled quality score ≥ 20 and the average quality ≥ 15 for the 11 bp surrounding the putative SNP.

SNPs were manually selected based on their distribution on the peach genome. SNPs surrounded by repetitive sequences and/or located within a stretch of bases identical to that of the SNP itself (i.e. a short stretch of A in an A/G SNP) were avoided. The surrounding sequences (about 150 bp/side) were obtained from the peach Gbrowse available on the IGA website [60] and blasted against the v1.0 peach genome at GDR [61] to verify their uniqueness. Only unique SNP-surrounding sequences were used to design the assays (a locus specific primer pair and a single-base extension primer or probe for each SNP), and combine them in multiplex reactions (hereafter called iPlex) by the software Mass ARRAY Design 3.1.

All locus-specific PCR primers and probes were blasted against the peach genome to further verify their specificity. Only the SNPs that passed all these quality checks were retained for further analyses.

A total of ten iPlex were designed to attain the optimal genome coverage, including two iPlex (steps 9 and 10) with SNPs selected in specific uncovered regions, or where SNPs previously tested were not useful. Information about the primers used for the genotyping is listed in Additional file 1: Table S2.

Genotyping in the CxA progeny was performed using iPLEX Gold technology [62] and Mass ARRAY high-throughput DNA analysis mass spectrometry (Sequenom, Inc) at the Centre for Applied Biomedical Research (CRBA) of Bologna.

The Sequenom data for each SNP were first verified by checking the heterozygosity of the CxA F_1_ parent and its consistency with the two grandparents ‘Contender’ and ‘Ambra’. All the SNP data were integrated with the dataset of 31 SSR markers genotyped following Eduardo et al. [51], and then analyzed by JoinMap 3.0 software [63] with the default parameters and the Kosambi [64] mapping function. Linkage groups were established at LOD value (independent LOD score) higher than 10.

All these SNPs were also tested on the BIN set of the TxE mapping progeny.

### IPSC 9 K SNP array genotyping and mapping

The IPSC 9 K SNP array [31] was used to genotype 242 individuals of the PxF and 62 of the MDxSD progenies, using the Illumina Infinium II design probes, and the dual color channel assay (Infinium HD Assay Ultra, Illumina). SNP genotypes were scored with the Genotyping Module of the Genome Studio Data Analysis software (Illumina, Inc.). SNPs with a GeneTrain score ≥0.4 and less than 10% missing data were retained. Allele segregation was also checked and SNPs showing unexpected segregations (as, for example, with parents homozygous for the same allele) or unexpected genotype classes (with respect to the parental genotypes), were inspected using Genome Studio. If possible they were re-clustered using the “define cluster” function. Those still showing missing or unexpected classes were discarded.

Linkage analysis and map construction were performed with JoinMap 4.1 [65] using the CP and BC_1_ population types for PxF and MDxSD, respectively. Linkage groups were established at LOD value (independent LOD score) higher than 10 as described above. The Multipoint Maximum Likelihood mapping algorithm was used with the default parameters. Kosambi units [64] were used; for the CP population type, Haldane map distances were manually converted in Kosambi units using the formula provided in JoinMap 4.1 manual.

### Map-sequence integration

The original raw version of the Peach v1.0 assembly, post filtering organelle, repetitive, and small scaffolds (< 1 kb) sequences, was used for the new map-sequence integration. Markers were placed on the WGS scaffolds using two methods as reported in Verde et al. [20]. SSR and SNP markers having primer sequences (i.e. the MASSARRAY developed markers) were placed using three successive rounds of electronic PCR (e-PCR [66]) with *N* = 0, *N* = 1 and *N* = 3. Markers with a known sequence, including RFLP (Restriction Fragment Length Polymorphism) and SNP markers or SSRs whose primers had not been found with the three rounds of ePCR, were placed with BLASTN. The additional breaks were made in regions of low BAC/Fosmid coverage and the broken sequences reordered according to the new maps. The mapped WGS scaffolds were joined as described in Verde et al [20] to form 8 pseudomolecules (Pp01 to Pp08). Each map join is denoted by 10.000 N bps.

In this work, to avoid confusion between v1.0 and v2.0 releases, scaffolds composing the Peach v1.0 assembly were named and are hereafter referred to as “Scaffold_##”. The 40 WGS scaffolds included in the 8 v1.0 pseudomolecules were named as “Scf_##”. The WGS scaffolds composing the v2.0 pseudomolecules (Pp01 to Pp08) were named as “Super_##”.

### Lovell DH resequencing

Resequencing of the ‘Lovell’ double haploid (PLov2-2n) was performed using the MiSeq Illumina platform. Paired-end reads (43x 2x250 bp, 600 bp insert size and 21x 2x250 3 kb and 6 kb insert size Additional file 1: Table S3) were assembled with the AbySS software [67] after quality checking and filtering. The resulting contigs were used to patch gaps in the Peach v1.0 assembly after the new breaks and joins described above were applied (hereafter referred to as “modified v1.0 assembly”). Contigs were aligned to the repeat masked modified v1.0 assembly using BLAT [68]. Contigs whose ends aligned to either side of a gap, with at least 1/3 of the contig length anchoring to the edges of the gap at ≥95% identity, were used to patch the gap. Sequence and quality scores were then integrated into the v1.0 modified assembly. Finally, homozygous SNPs and indels were corrected using ~43x Illumina reads. Reads were aligned using BWA [69] and variants (SNPs and indels) called using the standard GATK pipeline [70] including base quality score recalibration, indel realignment, and duplicate removal.

### Physical vs genetic distance comparison and identification of centromeric regions

MareyMaps were obtained, for each mapping progeny, by plotting the genetic positions of molecular markers (in centimorgans, cM) against their physical position on the Peach v2.0 (in Megabase pairs, Mb) [71]. Cumulative recombination curves for each chromosome were estimated using the cubic spline interpolation method with default parameters and the cross-validation type present in the MareyMap package. The recombination value per position was obtained calculating the slope per markers and their curves were plotted for each chromosome.

In order to identify the putative centromeric region of each chromosome, regions displaying the lowest recombination rate, as highlighted by the MareyMaps, were manually checked on the Peach v2.0 using the JBrowse available on Phytozome [57] for the absence of transcripts and the abundance of repeated elements associated with centromeric and pericentromeric regions. Sequences retrieved from Neumann et al. [72], representing a catalog of plant repeated elements associated with centromeric regions, were aligned with BLASTN [57] to the peach genome assembly. Sequences aligning within the putative peach centromeric regions (1 Mb of sequence around the predicted centromere), with at least 63% of identity and an e-value greater than 4 x 10^−16^, were retained.

Recombination frequency was compared among all progenies by multiple comparison statistics implemented in PAST 2.12 [73]. For each linkage map used in this study (TxE, CxA, and PxF), recombination rate was estimated at individual whole-chromosome scale as the ratio between genetic (cM) and physical (Mb) distances. One-way analysis of variance (ANOVA) was applied with Tukey’s pairwise *post-hoc* test. The Levene’s test for homoscedasticity and the Shapiro-Wilk test for normal distribution were also applied to check the assumptions for the applicability of the ANOVA. In the case of violation, the non-parametric Kruskal-Wallis test was applied instead, with the Mann-Whitney pairwise *post-hoc* comparison and the Bonferroni correction.

## Results and Discussion

Four linkage maps were used to improve the peach genome. TxE and CxA were already available [45, 51] and were enriched using a targeted approach. Another map was the result of a *de-novo* mapping of the PxF progeny [52] obtained using the IPSC 9 K SNP array [31] and tripling the mapping progeny size. Finally, the MDxSD map, also obtained with the IPSC 9 K SNP array, was used to specifically address some inconsistencies at the top of linkage group 6 (LG6).

### Targeted SSR identification and mapping in TxE

A total of 111 SSRs (RPPG set) were identified in the Peach v1.0 assembly and primers were designed and then tested in the TxE BIN set (Additional file 1: Table S1). Twenty markers were developed on the major unmapped scaffolds, 14 were individuated within the randomly oriented scaffolds and 77 were identified within the 24 uncovered scaffold ends. Thirty-eight out of the total were monomorphic in TxE and 73 were BIN mapped (65.8% of polymorphism; Additional file 1: Table S1). Out of 56 microsatellites falling within genic regions, 40 (71.4%) were polymorphic. Three polymorphic markers (RPPG14-003, RPPG16-002, RPPG5-005) were mapped in TxE, and found in successive analysis to have their primer pair on the same flanking side of the microsatellite region. These three markers were retained and reclassified as indel markers. These incidental length polymorphisms in the TxE interspecific progeny reflect the different genomic structure of the closely related almond and peach species. The same length variation was observed in six out of the seven *Knox* genes whose fragment size indicated differences between the two parents ranging from 2 bp to 20 bp in length [74]. A much higher level of polymorphism (89.2%) with RFLPs in the TxE progeny had been already observed (Dettori, unpublished results) in comparison to that (28.4%) of the intraspecific PxF progeny [52]. Thirty-two well-spaced SSRs, out of the 73 polymorphic ones, were mapped in the whole TxE progeny to resolve orientation, misassembly, or ordering discrepancies.

In the preliminary steps of the peach genome assembly 54 SSRs [47, 48, 75] targeting specific regions had been mapped in the whole progeny (Additional file 1: Table S4). In addition, seven markers (SNPs and indels) targeting the peach *KNOX* genes [74] had also been included. The final map (Additional file 2: Figure S1, Additional file 1: Table S4) is composed of 655 markers and covers 511.3 cM. Three hundred and twenty-nine markers, having sequence information associated, were used for the Peach v2.0 map-sequence integration. They cover 472.7 cM (92.3% of the total genetic distance in TxE) and 220 Mb (97.5% of the v2.0 pseudomolecule length, Table 1). Only four gaps larger than 10 cM are present. In addition, 449 ROSCos BIN mapped markers [76] and four SNPs from the *Prunus-Malus* consensus sequence [77] were included in this study and integrated with the WGS scaffolds. Furthermore, 53 SNPs from the CxA F_1_ parent and 41 SSRs isolated in this study (RPPG set) were also BIN mapped. In total 1,224 TxE markers were integrated within the assembly, 895 BIN mapped (348 used in Peach v1.0 and 547 added in this study), and 329 mapped in the whole progeny (Table 1, Additional file 1: Table S4).

**Table 1:** Anchoring statistics of the Peach v2.0 assembly

		Chromosome (LG) Pseudomolecule								
	Mapping progeny	1	2	3	4	5	6	7	8	Total
Number of markers integrated^a^	TxE	256 (186)	143 (101)	155 (113)	120 (83)	123 (90)	159 (121)	143 (107)	125 (94)	*1,224 (895)*
	PxF	269	207	292	408	153	219	224	202	*1,974*
	CxA	29	24	36	42	12	29	25	28	*225*
	*Total*	*554*	*374*	*483*	*570*	*288*	*407*	*392*	*355*	*3,423*
Number of scaffolds anchored	TxE	10	9	10	7	5	8	4	4	*57*
	PxF	9	8	8	7	5	7	4	3	*51*
	CxA	9	8	9	6	3	9	4	4	*52*
	*Total*	*10*	*9*	*10*	*7*	*5*	*9*	*4*	*4*	*58*
Genetic distances covered (cM)	TxE	77.5	42.7	44.1	51.7	47.6	81	70.6	57.5	*472.7*
	PxF	117.4	70.9	69.9	69.3	62.1	81.1	67.3	67.6	*605.6*
	PxF F_1_	139.8	88.1	71.9	76.7	59.1	91	63.6	68.4	*658.6*
	PxF recurrent	53.7	31	--	44.2	27.6	45.9	19.6	10.4	*232.4*
	CxA	98	36.3	67.7	64.5	36.5	79.9	62.3	64.4	*509.6*
Physical distance in bp covered with the integrated markers and *(%)* of Peach v2.0 pseudomolecules	TxE	47,190,243 *(98.6)*	29,794,491 *(98.0)*	27,174,422 *(99.3)*	24,974,520 *(96.6)*	17,300,580 *(93.5)*	30,384,999 *(98.8)*	21,009,142 *(93.8)*	22,199,033 *(98.3)*	*220,027,430 (97.5)*
	PxF	46,854,330 *(97.9)*	29,975,524 *(98.6)*	26,162,111 *(95.6)*	25,167,755 *(97.4)*	17,989,526 *(97.3)*	29,985,579 *(97.5)*	22,201,468 *(99.2)*	20,421,932 *(90.5)*	*218,758,225 (96.9)*
	PxF F_1_	46,854,330 *(97.9)*	29,652,167 *(97.5)*	26,162,111 *(95.6)*	25,167,755 *(97.4)*	17,989,526 *(97.3)*	29,209,364 *(94.9)*	20,322,548 *(90.8)*	20,087,434 *(89.0)*	*215,445,235 (95.5)*
	PxF recurrent	39,665,700 *(82.9)*	22,115,897 *(72.7)*	--	19,074,112 *(73.8)*	5,684,854 *(30.7)*	26,868,520 *(87.3)*	3,306,229 *(14.8)*	3,031,158 *(13.4)*	*119,746,470 (53.1)*
	CxA	45,955,086 *(96)*	19,656,032 *(64.6)*	27,246,203 *(99.6)*	25,053,083 *(96.9)*	7,348,324 *(39.7)*	29,848,481 *(97.0)*	21,600,273 *(96.5)*	22,073,557 *(97.8)*	*198,781,039 (88.1)*
*Total No of bases anchored (bp)*		*47,851,208*	*30,405,870*	*27,368,013*	*25,843,236*	*18,496,696*	30,767,194	22,388,614	*22,573,980*	*225,694,811*

For each map and for each chromosome the number of markers, number of anchored scaffolds, genetic and physical distance covered with the integrated markers and the total number of anchored bases are reported

^a^In brackets the BIN mapped markers in TxE

### Targeted SNP identification and mapping in CxA

From the resequencing of the CxA F_1_ parent, 265 SNPs have been developed and included in a total of ten iPLEX assays, with an average number of 26.5 SNP each iPlex. Among the tested SNPs, 194 were useful for mapping while 71 were not: of these, 49 were monomorphic (38 showing only one allele and 11 being heterozygous in the whole progeny), 12 showed only two over three expected genotypes and ten presented more than 25% of missing data. The latter group also included five markers with no amplification in the whole progeny, possibly due to assay failure.

The fraction of scorable polymorphic SNPs ranged from 52.2% to 86.2% in different iPLEX assays, with an average success rate of 73.2% (Additional file 1: Table S5). The relatively negative result on iPLex 9 and 10 is likely due to forcing the iPlex design to develop markers in target regions.

The observed SNP calling efficiency was evaluated by comparing our results with those obtained by Verde et al. [20], which used more stringent parameters for SNP calling. This *a posteriori* analysis showed that 40.8% of the non-polymorphic SNPs were false positives (29 markers out of 71) but at the same time five true SNPs over the 194 mapped ones (7%) would have been lost using the more stringent conditions (false negatives). Finally, with the more stringent parameters, the total efficiency of SNP design would have increased from 73.2% to 80.1% (Additional file 1: Table S5).

The CxA map was first obtained with 31 SSRs on 169 F_2_ progeny [51]. To improve the chromosome-scale assembly the number of progeny was increased to 305. The additional individuals were genotyped with SSR markers, adding 194 targeted polymorphic SNPs to the map. Fifteen of these SNPs were already linked on LG4 to a candidate gene controlling maturity date in peach [78]. Another set of twelve SNPs on LG5 surrounding the nectarine *G* locus had been described [79]. A total of 20 SNPs were identified on unmapped scaffolds, ten on the randomly oriented scaffolds and 15 in putative chimeric regions. Fifty-three of these SNPs were also BIN mapped in TxE, as already described in the previous paragraph. The final CxA map (Additional file 2: Figure S1; Additional file 1: Table S6) includes 225 markers (SSRs and SNPs) corresponding to 211 unique genetic positions, covering 509.6 cM with only four gaps larger than 10 cM. All the pseudomolecules are almost completely covered (198.8 Mb, 88.1% of the v2.0 pseudomolecule length, Table 1), with the exception of Pp02 lacking the bottom portion (about 10 Mb, ~35% of the total length) and Pp05 missing the upper portion (about 11 Mb, ~60% of the total length).

### IPSC 9 K SNP array mapping in PxF

The 242 trees of the PxF progeny were genotyped with the IPSC 9 K SNP array [31]. Out of the 8,144 placed on the array, a total of 3,399 polymorphic SNPs (41.7%) were identified; 1,669 SNPs were informative for the F_1_ parent (segregating in a 1:1 ratio), 641 were informative for both parents (1:2:1 ratio) and 1,089 were informative for the recurrent parent (1:1 ratio). Of the three types of segregation, only the first two were used for the map-sequence integration because the unequal recombination frequencies between the two parents of the cross inhibited efficient joining of their genetic information. In fact, the recurrent parent (IF7310828) displays a marked reduction in recombination frequency in comparison to that of the F_1_ parent (1.941 vs 3.057 cM/Mb, on average, Table 2, Additional file 1: Tables S7, S8, S9). As a consequence, the map-sequence integration was unreliable due to the heavily skewed order of markers in regions with strong differences in recombination frequencies between the two parents. Moreover, the distribution of the informative SNPs for the recurrent parent was uneven across the genome resulting in a fragmented linkage map with 12 groups. In fact, five groups were split in two groups each (LG2, 5, 6, 7 and 8), one was completely missing (LG3) resulting in a reduced genome coverage (119.7 Mb, 53.1% of the Peach v2.0 pseudomolecule length, Table 1, Additional file 1: Table S9). The inspection of the IPSC 9 K SNP array markers segregating in the recurrent parent highlighted that they did not give additional information for integration except for two contiguous minor scaffolds (Super_23 and Super_456, 1.5 Mb in total) found in the upper part of the Pp01. Seven markers located in these two scaffolds informative for the recurrent parent, were selected and integrated into the map. The presence of bridge markers in that region (about 10 Mb) segregating in a 1:2:1 ratio and the low number of 1:1 markers informative for the F_1_ parent (5 out of 52) enabled a reliable integration. When a single SNP was mapped in a linkage group that differed from the expected placement from the array information, and no further evidence of misassembly was observed in the same genetic region (i.e. SNP not located at the scaffold terminals), this marker was deemed as putatively duplicated (from putative paralogous genes) and excluded.

**Table 2:** Genetic/physical ratio (cM/Mb) for each map and each chromosome

LG-Pp^a^	TxE	PxF	PxF F_1_	PxF recurrent	CxA	WxB	DvsS
1	1.642	2.506	2.984	1.354	2.133	2.261	1.858
2	1.433	2.365	2.971	1.402	1.847	2.141	1.435
3	1.623	2.672	2.748	--	2.485	2.340	2.174
4	2.070	2.754	3.048	2.317	2.575	2.544	2.384
5	2.751	3.452	3.285	4.855	4.967	3.777	2.672
6	2.666	2.705	3.115	1.708	2.677	2.393	1.770
7	3.360	3.031	3.130	5.928	2.884	3.003	1.930
8	2.590	3.310	3.405	3.431	2.918	2.775	1.812
Total	2.148	2.768	3.057	1.941	2.564	2.553	1.954

^a^ *LG* Linkage Group, *Pp* Pseudomolecule

After filtering, we mapped 1,974 SNPs in total (1,566 segregating in a 1:1 ratio informative for the F_1_ parent, 401 segregating in a 1:2:1 ratio and 7 informative for the recurrent parent) corresponding to 567 unique genetic loci. The map (Additional file 2: Figure S1, Additional file 1: Table S7) covers 605.6 cM (corresponding to 218.8 Mb, 96.9% of the v2.0 pseudomolecule length, Table 1) with only one gap larger than 10 cM.

### Integration of unmapped scaffolds in Peach v2.0 pseudomolecules

One hundred and ninety-four scaffolds (8.7 Mb in total, 4% of the total assembly size), had not been included in the eight peach v1.0 pseudomolecules. To anchor the larger unmapped scaffolds (> 300 kb) in Peach v1.0, we first used the TxE map. Twenty SSRs (Additional file 1: Table S1) were targeted in the unmapped portion of the peach genome and 17 polymorphic ones (11 fully mapped) enabled anchoring of the ten major scaffolds (v1.0 Scaffold_9 to Scaffold_18) and fixing the orientation for two of them (v1.0 Scaffold_9 and Scaffold_10, 2.1 Mb and 851 kb, respectively; Additional file 2: Figure S1, Additional file 1: Tables S3 and S9). The same was done using the CxA map (Additional file 1: Table S2). This map (Additional file 2: Figure S1, Additional file 1: Table S6), in addition to being from an intraspecific cross, was obtained with a large mapping progeny (305 plants) providing a fine estimation of the recombination frequencies even at a small scale (about 100 kb). With this approach, we were able to confirm the anchoring of the ten major scaffolds (>300 Kb) and anchoring of an extra minor scaffold (v1.0 Scaffold_36, 23 kb in size). The high resolution of the CxA map also enabled the ordering of two contiguous small scaffolds (v1.0 Scaffold_12 and Scaffold_16) on pseudomolecule 2, that in TxE were unordered and unoriented, and fixing the orientation of five scaffolds (v1.0 Scaffold_9 Scaffold_10, Scaffold_11, Scaffold 12 and Scaffold_15, 4.9 Mb in total). Only 19 markers of the IPSC 9 K SNP array were located in the unmapped portion of the genome and six of them were polymorphic in PxF. The position of five of the ten major scaffolds previously mentioned (v1.0 Scaffold_10, Scaffold_12 Scaffold_13, Scaffold_14, and Scaffold_17) was confirmed using these markers.

Together, these analyses positioned 11 unmapped scaffolds on the v2.0 pseudomolecules (Additional file 1: Table S10). They cover 7.2 Mb of sequence (3.2% of the total assembly); five of them were also orientable (4.9 Mb, 2.2% of the total assembly; Additional file 1: Table S10).

### Ordering and orientation of Peach v1.0 randomly oriented sequences

Ten mapped scaffolds (15.8 Mb) of the Peach v1.0 genome were placed with random orientation due to the lack of recombination among markers or because they were anchored with only one marker. To resolve the orientation of these scaffolds, previously BIN mapped markers, located by the ends of the non-oriented scaffolds were mapped in the whole TxE progeny. For scaffolds where no BIN mapped markers were available, 14 SSR primer pairs (Additional file 1: Table S1) were designed towards the end, tested on the TxE BIN set and the most physically distant polymorphic markers (8 SSRs) were mapped in the whole progeny. In this way, five integrated scaffolds (v2.0 Super_23, Super_25, and Super_10 on Pp01, Super_ 20 on Pp02 and Super_19 on Pp05, Additional file 2: Figure S1, Additional file 1: Tables S3 and S9) representing 10.4 Mb of sequence were oriented along the pseudomolecules. Moreover, the attempt to orient a scaffold on Pp02 (v2.0 Super_20) revealed that it was incorrectly placed along the pseudomolecule due to a mismapped marker (CPDCT044) in TxE. This scaffold was correctly positioned at the top of the Pp02. The higher genetic resolution of CxA map (Additional file 2: Figure S1, Additional file 1: Tables S5 and S9) confirmed the position of Super_20 at the top of Pp02 and the correct orientation of a major scaffold at the bottom of Pp011 (v2.0 Super_10). It also rectified the orientation of two wrongly oriented scaffolds due to mapping artifacts in TxE: one on top of Pp07 (v2.0 Super_11, 4.8 Mb) and the other in the middle portion of Pp08 (v2.0 Super_15, 2.9 Mb). The high density and resolution of the PxF map further confirmed the orientation of the above-mentioned scaffolds and enabled the correct orientation of three other scaffolds in Peach v1.0 (v2.0 Super_451 on Pp03 and Super_26 and Super_29 on Pp04; 2.9 Mb). In a region of Pp03 (12–17.6 Mb), indicating high recombination frequency suppression, five scaffolds (v2.0 Super_451, Super_18, Super_27, Super_31, Super_32) were ordered with low probability in TxE. The higher resolution of CxA and PxF helped to resolve uncertainties (i.e. the position of Super_18 embedded between Super_31/Super_32 and Super_451/Super_27). However, the orientation of Super_31 and Super_32 was indeterminate in v2.0 since the corresponding markers cosegregated in all maps. Moreover, it was not possible to have certainty of the order of Super_451 and Super_27 located in the same region (Pp3, 15.3–17.6 Mb). In fact, markers on those scaffolds were ordered in TxE with low probability (alternative positions to the accepted one were only slightly less likely, with a difference in log-likelihood of 0.37, i.e. 2.34 folds less likely). In CxA and in PxF only one of these two scaffolds was anchored in each map (Super_451 in PxF and Super_27 in CxA) giving no additional information on their order. For these scaffolds, the Peach v1.0 order, based on TxE, was retained. However, recently published maps [80, 81] obtained using the IPSC 9 K SNP array [31] have enabled verification of their order and orientation. In particular in the MxR_01 map [80] two SNPs (SNP_IGA_336437 and SNP_IGA_339719) mapping on Super_27 at 39.5 and 41.1 cM, respectively (Peach v2.0 position at 17,026,649 and 17,569,078 nt, respectively) and other four (SNP_IGA_326457, SNP_IGA_328528, SNP_IGA_331373 and SNP_IGA_333074) mapping on Super_451 at 42.8, 45.9, 49.2 and 50.7 cM, respectively (Peach v2.0 position 15,586,851, 15,899,181, 16,311,538 and 16,634,203 nt, respectively) revealed that the order established in Peach v2.0 is incorrect and will be inverted in a future release. This is also confirmed by the DvsS map [81] in which two SNPs (SNP_IGA_338615 at 17,411,354 nt on Super_27 and SNP_IGA_325296 at 15,442,995 nt on Super_451) were mapped at 31 and 32 cM, respectively. The map obtained by Sánchez et al. [80] confirmed the orientation of Super_451 established only by PxF map and v2.0 orientation of Super_27 was shown to be correct, though it was anchored by only one marker in TxE and CxA.

Together, all the ten scaffolds randomly oriented in Peach v1.0 and two wrongly placed (summing up 23.6 Mb of sequence, 10.4% of the Peach v2.0 total length) were correctly oriented in Peach v2.0 pseudomolecules. Currently, only six minor scaffolds (Super_31, Super_32, Super_34, Super_36, Super 35, and Super_54, Additional file 1: Table S10) summing up 2.4 Mb are randomly oriented in this release. Moreover, only a known mis-order (Super_27 and Super_451) and a random order (Super_31 and Super_32) in the central part of chromosome 3 are still present in Peach v2.0 (Additional file 1: Table S10). These will be rectified in a future release of the peach genome.

### Scaffold ends checking and correction of misassembled sequences

Within the 40 scaffolds composing the Peach v1.0 pseudomolecules, 24 terminals were not covered with molecular markers for at least 500 kb of their length, 13 having an uncovered portion larger than 1 Mb with the largest one being of 3.1 Mb. These regions are potential sites of misassembled sequence. To check scaffold consistency, SSR and SNP markers were developed in the distal part of these uncovered regions. Markers mapped in TxE and CxA maps (48 SSRs and 15 SNPs, respectively) helped to reveal five out of these 24 uncovered scaffold ends as sites of misassembly: three in pseudomolecule 4, one in pseudomolecule 3 and one in pseudomolecule 7. On pseudomolecule 4, there were two scaffolds bearing two different chimeric regions, resulting in six pieces in total that needed to be relocated on different chromosomes. For this purpose, the two most distant polymorphic markers in each chimeric region were mapped in the whole TxE progeny in order to locate and orient the new broken scaffolds within the peach pseudomolecules. A particular case occurred in the v1.0 integrated scaffolds Scf_450 and Scf_451 located on pseudomolecule 3 and pseudomolecule 7, respectively. They originated from a chimeric scaffold that had been broken in a wrong position in the Peach v1.0 assembly due to insufficient marker coverage within the putative chimeric region (about 1.2 Mb gap). In v2.0, with the help of the new mapping data, we refined the breakpoint, so that 385 kb from v1.0 Scf_450 in pseudomolecule 3 were re-joined to the formerly broken Scf_451 to form the new Super_452 on v2.0 Pp07 (Additional file 1: Table S10).

The whole genome mapping approach of the PxF map confirmed all of the chimeric scaffolds and enabled identification of two other cases of misassembly on the top of v1.0 pseudomolecule 6. In this region two scaffolds (Scf_26 and Scf_457, 14.8 Mb total sequence, Additional file 1: Table S10) were chimeric and needed to be broken in one point each. Three of the broken portions, 4.6 Mb of sequence (Super_447, Super_464 and Super_446, 602 kb, 3.3 Mb and 709 kb, respectively), had to be rearranged within the same chromosomal region (Additional file 2: Figure S1, Additional file 1: Tables S6, S7, S8, S9). To support the rearrangements at the top of v1.0 pseudomolecule 6, we used additional information from the MDxSD map. In this map, LG6 is composed of 153 SNP markers for a total of 27 single genetic positions covering a genetic distance of 54.9 cM with an average of 2.03 cM between markers and a major gap of 6.5 cM (Additional file 2: Figure S1, Additional file 1: Table S11). The total physical distance covered amounts to 29,6 Mb (96.1% of Pp06 length). Twenty-one MDxSD markers mapped in the 4,6 Mb region (spanning 9.7 cM) confirm the order of the three broken scaffolds highlighted by PxF and the orientation of two of them (Super_447 and Super_464). Without high resolution and high-density linkage maps highlighting discrepancies in this 4.6 Mb region (41 markers in PxF and 21 in MDxSD), this problem could not have been solved since the TxE and CxA maps have low marker density in that region. Scaffold ends were also checked using additional information from recently published linkage maps [80–82]. In the current assembly, only 3 scaffold ends larger than 500 kb are still not covered with markers, all of them lying in highly repeated centromeric regions, with the largest being a 594 kb stretch of sequence (Additional file 1: Table S10).

In total, 10.4 Mb (4.6% of the total assembly) of sequence from Peach v1.0, were relocated in their correct chromosomal positions with the correct orientation.

### Base accuracy and contiguity improvement through reference accession resequencing

The ‘Lovell’ double haploid (PLov2-2n) was resequenced from 5 libraries (Additional file 1: Table S3), producing 73,881,213 paired-end reads, corresponding to 43-fold paired end fragment sequence coverage (2x250, 600 bp insert size) and 21-fold mate pair coverage (2x250, 3 kb and 6 kb insert size). This set of reads was assembled using ABySS (v1.3.6) [67], producing 30,131 scaffolds greater than 500 bp for a total of 180.7 Mb of sequence. Scaffolds produced in the ABySS assembly were broken into contigs, and a total of 206 contigs representing 3.2 Mb of sequence were used to close 212 Peach v1.0 gaps, with a gain of 25,199 kb (Table 4). The overall contiguity was improved, with the total number of contigs in Peach v2.0 decreased by 7.5% dropping from 2,730 to 2,525 and the contig L50 increased by 19.2% (from 214.2 kb to 255.4 Kb). Finally, 859 homozygous SNPs and 1,347 indels were corrected using ~43x paired end fragment Illumina reads (Table 3).

**Table 3:** Summary of gap patching and indel and SNP correction

Pseudomolecules	No. of contigs	No. of gaps closed	Gap bases patched	Initial contig length	Post gap-patching contig length	Bases gained	Indels corrected	SNP corrected
Pp01	36	36	6,820	47,412,656	47,417,444	4,788	269	143
Pp02	27	27	6,884	29,982,897	29,985,295	2,398	185	117
Pp03	29	30	4,831	27,022,361	27,022,947	586	167	128
Pp04	24	24	3,895	25,545,546	25,549,276	3,730	133	132
Pp05	19	21	10,824	18,291,031	18,295,669	4,638	110	58
Pp06	25	26	9,635	30,419,305	30,423,361	4,056	197	117
Pp07	29	30	7,501	22,049,797	22,053,146	3,349	157	75
Pp08	17	18	3,485	22,391,144	22,392,798	1,654	129	89
*Totals:*	*206*	*212*	*53,875*	*223,114,737*	*223,139,936*	*25,199*	*1,347*	*859*

### Chromosome-scale assembly and comparison to other published genomes

**Fig. 1:**
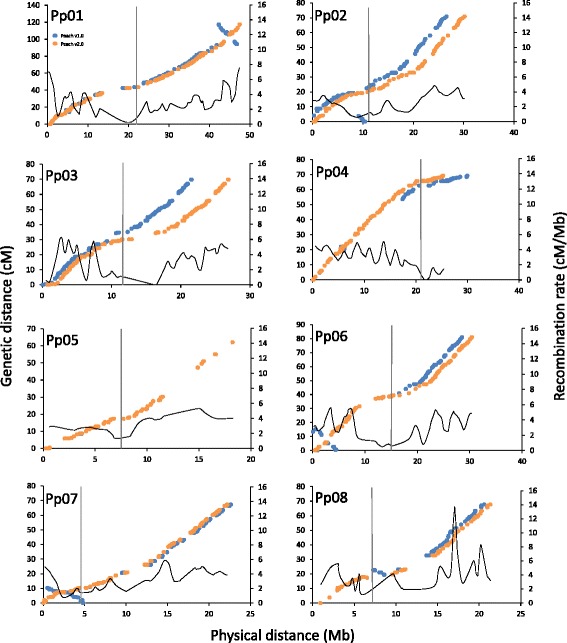
Plots of genetic-by-physical distances (MareyMap). Comparison of v1.0 and v2.0 physical distances (Mb, in the horizontal axis) and PxF genetic distances (cM, in the vertical axis). Dots represent the mapped markers. The vertical bars indicate the putative position of the centromere. The solid line represents the recombination rate plotted along the 8 pseudomolecules

In the new map-sequence integration, a total of 3,423 markers (Table 1) from three linkage maps were used in the integration of the original raw peach assembly obtained prior to the build of Peach v1.0 pseudomolecules: 1,224 markers were integrated in TxE (895 of them BIN mapped), 1974 in PxF and 225 in CxA (Table 1). The improved TxE map (Additional file 2: Figure S1, Additional file 1: Table S4) enabled the anchoring of 57 scaffolds (225,7 Mb; 99.2% of the peach genome) and orienting of 40 scaffolds (207.5 Mb; 91.3% of the peach genome; Additional file 1: Table S10). The CxA map (Additional file 2: Figure S1, Additional file 1: Table S6) anchored 52 scaffolds (211.1 Mb; 92.8% of the assembled sequences) and oriented 33 (178.1 Mb; 78.3% of the assembled sequences; Additional file 1: Table S10). The PxF map (Additional file 2: Figure S1, Additional file 1: Table S7) anchored 51 scaffolds (220.8 Mb; 97.1% of the assembled sequences) and oriented 44 (215.6 Mb: 94.8% of the assembled sequences; Additional file 1: Table S10). Finally, 153 SNPs mapped in MDxSD on LG 6 were instrumental in confirming some faults at the top of Pp06 (Additional file 1: Table S11). Important amendments were made in the v2.0 assembly including the portion of mapped and oriented sequences and the identification and correction of discernible misassemblies. A visual comparison between the two versions of the peach genome (v1.0 vs v2.0) is shown with the MareyMap plot (Fig. 1). High-quality assemblies are those in which the plots are characterized by a monotonically increasing function. Both Peach v1.0 and v2.0 show a general increasing tendency, however, in few v1.0 regions the function decreases highlighting this release faults (misordered, misoriented or local misassembled scaffolds such as in Pp01, Pp06, Pp07). Furthermore, differences in the physical length between the two assemblies, observed in all but Pp05 pseudomolecule, reflect the inclusion of previously unmapped scaffolds and the correction of inter-pseudomolecule misassemblies (Additional file 2: Figure S1 and Additional file 1: Table S10).

The total number of major WGS scaffolds (> 1 kb) after filtering and scaffold breaking and prior to pseudomolecule construction was 241, spanning 226,911,381 bp with a scaffold N50/L50 of 10/7.3 Mb (Additional file 1: Table S12). The final chromosome-scale assembly is arranged in 191 stretches of non-contiguous sequences (8 pseudomolecules and 183 scaffolds) spanning 227,411,381 bp with a contig length of 224,638,928 bp and a scaffold contig coverage of 98.8% (Additional file 1: Table S113). Fifty-eight scaffolds spanning 225.7 Mb of sequences were integrated into the 8 Peach v2.0 pseudomolecules corresponding to 99.2% of the total sequence (Table 4, Additional file 1: Table S10). A comparison between the statistics of the two chromosome-scale assemblies, Peach v1.0 and Peach v2.0 is also shown in Table 4. Fifty-two scaffolds, summing up 223.3 Mb (98.2%), are correctly oriented in the new assembly (Additional file 1: Table S10). The unmapped portion of the genome comprises 183 scaffolds with a length of 1.7 Mb (0.8%) containing only 62 predicted genes [57]. Additional file 1: Table S10 resumes the integration of Peach v2.0 assembly, reporting the scaffolds mapped in the 8 pseudomolecules, their position in Peach v1.0 and Peach v2.0, the orientation information in all the maps and the size of uncovered scaffold ends.

**Table 4:** Summary statistics of the Peach v2.0 chromosome-scale assembly statistics and its comparison with the v1.0

	Peach v2.0	Peach v1.0
Number of scaffolds	191	202
Number of contigs	2,525	2,730
Scaffold sequence	227.4 Mb	227.3 Mb
Mapped scaffold sequence	225.7 Mb (99.2%)	218.4 Mb (96%)
Oriented scaffold seqeuence	223.3 Mb (98.2%)	194.6 Mb (85.6%)
Contig sequence	224.6 Mb	224.6 Mb
Scaffold N/L50	4/27.4 Mb	4/26.8 Mb
Contig N/L50	250/255.4 kb	294/214.2 kb
Number of scaffolds > 50 KB	11	21
% main genome in scaffolds > 50 kb	99.4%	99.4%

Table 5 reports a comparison among some of the major plant genome assemblies released to date. The table was adapted and updated from Verde et al.[20] with newly published genomes and the updated genome releases (soybean, poplar, *Brachypodium*, *Sorghum*, *Physcomitrella*) available on Phytozome [57]. Among the WGS assemblies, Peach v2.0 displays one of the largest portions of sequences mapped on chromosomes (99.2%). The scaffold N50/L50 (10/7.3 Mb), prior to pseudomolecules build, shows the best values among those available. Chromosome-scale N50/L50 (4/27.8 Mb) is comparable with other chromosome-scale assemblies. If we exclude the finished genomes (rice and *Arabidopsis*) and those almost finished (*Brachipodium* and *Sorghum*), the peach genome shows one of the best contig N50/L50 (250/255.4 kb), including the recent WGS pineapple genome [39] (contig L50 126.5 kb) obtained combining the third generation single molecule long reads (PACBIO and Moleculo) with the NGS short reads (Illumina and 454). The long read sequencing in pineapple was instrumental in increasing the scaffold L50 metric from 91 to 640 kb and the contig L50 from 6.5 to 126.5 kb [39]. PACbio long reads were also useful to improve the assembly of the *A. thaliana* L*er* genome [42]; the integration of 17x PACbio long-reads with the Illumina short reads improved the scaffold L50 that increased from 4.1 Mb to 12.8 Mb. The long read single molecule sequencing technologies are playing an increasingly important role in sequencing projects. In fact, the bioinformatics efforts of the assembling procedure are assisted and empowered by the availability of sequence stretches entirely spanning the repetitive regions, which are a major contributor to gaps present in assemblies.

**Table 5:** Comparison of the peach genome to other published plant genomes

Genome release [Reference]	Coverage	Assembled scaffold sequence Mb	Mapped sequences Mb *(%)*	N50	L50 Mb	N50	L50 Mb	Contig N50	Contig L50 kb	Sequencing methods
				Scaffold WGS^a^		Scaffold Chr^b^				
Peach (*Prunus persica*) v2.0 [20]	8.47x	227.4	225.7 *(99.2)*	10	7.3	4	27.4	250	255.4	Sanger (WGS)
Apple (*Malus x domestica*) [10]	16.9x	598.3	528.3 *(88.3)*	80	2	--	--	16171	13.4	Sanger, 454 (WGS)
*Arabidopsis thaliana* ^c^ [7]	--	119.7	119.7 *(100)*	--	--	3	23.5	--	--	Sanger, (BAC by BAC)
Rice *(Oryza sativa*)^d^ [5]	--	382.2	382.2 (100)	--	--	6	30.8	--	--	Sanger, (BAC by BAC)
Soybean (*Glycine max*)^e^ v2.0 [88]	8.04x	955.4^f^	932.5 (97.6)	--	--	10	48.6^f^	1548^f^	182.8^f^	Sanger (WGS)
Poplar (*Populus trichocarpa*)^e^ v3.0 [3]	9.44x^f^	423^f^	388 (91.7)	--	--	8^f^	19.5^f^	206^f^	552.8^f^	Sanger (WGS)
Grape (*Vitis vinifera*)^e^ [4]	8.4x	467.5	290.2 (62.1)	--	2.1	14	13.9	2012	66.4	Sanger (WGS)
Papaya (*Carica papaya*)^e^ [89]	<3x	271.7	235 (86.5)	--	--	74	1.3	7109	10.6	Sanger (WGS)
*Brachypodium distachyon* v3.1 [90]	9.4x	271.9	271.1 (99.8)	--	--	3	59.3	--	22000^f^	Sanger (WGS)
*Sorghum bicolor* ^*e*^ v3.1 [91]	8.5x	697.6	625.6 (89.7)	--	--	6	62.4	--	1200^f^	Sanger (WGS)
*Selaginella moellendorffii* [92]	7x	212.6	-- (--)	38	1.7	--	--	515	119.8	Sanger (WGS)
*Physcomitrella patens* ^e^, v3.3 [93]	8.92x	466.7	-- (--)	86	1.7	12^f^	17.4^f^	312^f^	464.9^f^	Sanger (WGS)
Tomato (*Solanum lycopersicon*) [94]	25x	781.3	759.9 (97.3)	52	4.5	6	64.8	3641	55.7	Sanger, 454, Solid, Illumina (WGS)
Banana (*Musa paradisiaca*) [95]	20.5x	472.2	331.8 (70.3)	65	1.3	8	28.6	2113	43.1	Sanger, 454 (WGS)
Citrus (*Citrus x clementina*) [96]	6.97x	309.9	288.6 (93.1)	--	6.8	--	31.4	--	115.9	Sanger (WGS)
Watermelon (*Citrullus lanatum*) [97]	108.64x	353.5	330 (93.4)	42	2.4	--	--	3315	26.4	Illumina (WGS)
*Amborella trichopoda* [98]	30x	706	--	50	4.9	--	--	6448	29.4	Sanger, 454, Illumina (WGS)
*Medicago truncatula* [99]	--	328.9	297.1 (90.3)	53	1.27	4	38.9	--	--	Sanger, 454, Illumina (WGS, BAC by BAC)
Melon (*Cucumis melo*) [100]	13.52x	361.4	316.3 (87.5)	26	4.68	6^g^	17.7^g^	--	18.2	Sanger, 454 (WGS)
Coffee *(Coffea canephora*) [101]	30x	568.6	364.1 (64.0)	108	1.3	5^g^	32^g^	2290	51.1	Sanger, 454, Illumina (WGS)
Cotton (*Gossipium raimondii*) [102]	103.6x	775.2	567.2 (73.2)	95	2.3	--	--	4918	44.9	Illumina (WGS)
Pineapple *(Ananas comosus*) [39]	410x	381.9	315.8 (82.7)	--	0.64	13^g^	11.8	--	126.5	PACbio, Illumina, 454, Moleculo, (WGS)

^a^N50/L50 statistics of the WGS assembly prior to pseudomolecule build

^b^N50/L50 statistics of the chromosome-scale assembly

^*c*^ *Arabidopsis* assembly, obtained using BAC by BAC approach, represents the golden standard for plant genome. Statistics were calculated from TAIR10 release. (http://www.ncbi.nlm.nih.gov/mapview/stats/BuildStats.cgi?taxid=3702&build=9&ver=2)

^d^Rice assembly, obtained using BAC by BAC approach, represents the golden standard for plant genome. Statistics were calculated from IRGSP Releases Build 4.0 (http://rgp.dna.affrc.go.jp/IRGSP/Build4/build4.html)

^e^Data retrieved from Schmutz et al. [88]; they recalculated the original statistics to better match chromosome-scale assemblies

^f^Data from recent releases retrieved from Phytozome

^g^Data were recalculated based on the original statistics reported in the paper

We compared the statistics of the current peach release with the standards established by Chain et al. [43]. According to these standards, the Peach v2.0 assembly can be classified as “Improved High-Quality Draft” since actions have been taken in assigning almost the whole sequence to chromosomes, in solving discernible misassemblies, filling gaps to reduce the number of contigs and correcting base errors. Moreover, the annotation of the release (Peach v2.1a) has greatly improved gene completeness using a large amount of RNA-seq data, as well as the annotated repeats which include low copy repeats and a complete set of Helitron transposons. The number of gene models in v2.1a decreased to 26,873 (was 27,853 in v1.0) resulting also in less fragmented gene models. The average number of transcripts per gene model increased to 1.75 from 1.03 (47,089 transcripts in v2.1a vs 28,689 in v1.0). The annotation improvements, not described in this work, are briefly reported on Phytozome [57], GDR [61] and IGA [60] websites. For all these features the improved peach release (v2.0 assembly and v2.1a annotation) can be further classified according to Chain et al. [43] as an “Annotation-Directed Improvement”, making it a useful tool for genome comparison and evolutionary studies, including gene studies such as alternative splicing analysis and metabolic pathway reconstruction.

### Physical vs genetic distance comparison and identification of centromeric regions

The availability of the TxE, CxA and PxF linkage maps covering most of the peach genome enabled a chromosome-scale comparison of the recombination frequencies along the genome. In Fig. 1, Additional file 3: Figure S2, Additional file 4: Figure S3, and Additional file 5: Figure S4, MareyMaps are plotted together with a function describing the genetic/physical ratio (cM/Mb). Average physical/genetic distance ratios for each individual chromosome and cross were calculated (Table 2) to be 2.148 cM/Mb in the interspecific cross TxE and 2.564 cM/Mb and 2.768 cM/Mb in the intraspecific crosses CxA and PxF, respectively.

The MareyMap plots (Fig. 1, Additional file 3: Figure S2, Additional file 4: Figure S3 and Additional file 5: Figure S4), constructed for the three different mapping progenies, indicates the expected monotonic increase along each chromosome except for a flat region where a marked suppression of recombination can be observed in each pseudomolecule. The survey of these regions on Peach v2.0 JBrowse [57] revealed the almost complete absence of predicted genes and the abundance of repetitive elements. This concurrent evidence suggest that these are likely the centromeric regions (pointed out as a vertical bar in Fig. 1, Additional file 3: Figure S2, Additional file 4: Figure S3, and Additional file 5: Figure S4) which are known to be mainly composed of interspersed tandem repeats and retrotransposons. Despite the centromeres have highly conserved function during cell division, their DNA sequences are not conserved [83]. In particular, in plants, the centromeric satellite DNA repeats are species-specific. Recent studies suggest that these sequences underwent a rapid evolution revealing no sequence similarity among species diverged more than 50 Mya [84]. Other important components of the centromeric regions are represented by the retrotransposons directly involved in the centromere evolution and function [72]. A BLAST analysis against the peach genome was performed using a set [72] of 335 centromeric retrotransposon sequences belonging to 33 different plant species. In particular, eight plant centromere retrotransposons indicated similarity with the peach putative centromeric regions: three from *Medicago truncatula (*AC131249.44, AC147471.14, CT010572.8), two from *Pinus taeda* (AC241271.1, AC241322.1), one from *Picea glauca* (AF229251.1), one from *Beta vulgaris* (AJ539424.1), one from *Vitis vinifera* (AM426079.1)*.* These results strongly support that these regions are the peach centromeres. The accuracy of the Peachv2.0 assembly and the Peach v2.1a annotation enabled the positioning of the centromeric regions, which are generally very difficult to assemble due to their highly repetitive sequence composition. Linkage maps are of little use to assemble fragmented centromeric regions due to the suppression of recombination. As an example, the putative peach centromeric region in Pp03 (spanning Super_31 and Super_32, 12-13.2 Mb) indicated no recombination in the nearly 1000 meiosis analyzed in the three different mapping progenies, leaving the related scaffolds unoriented and unordered. The identification of putative centromeric regions for all of the eight Peach v2.0 chromosomes attests the completeness of the current peach genome assembly.

To verify whether there were significant differences in the recombination frequencies among the three maps, an analysis of variance was performed. Since the PxF map was obtained by map data of the two parents of the cross (the F_1_ and the recurrent IF7310828), the individual maps of the two parents were also considered in the analysis. Both the ANOVA and the non-parametric Kruskal-Wallis statistics were applied showing no significant difference among the five maps (*F = 0.9026 with p = 0.4733; Hc = 7.329 with p = 0.195*). However, due to the low coverage (referring to local rather than global recombination rates) of some linkage groups (Table 1) chromosomes with coverage lower than 50% of the total length (G3, G5, G7, G8 in PxF recurrent map and G5 in CxA) were excluded thus revealing significant differences with the ANOVA test (*F = 8.16; p = 0.00014*). Moreover, to enlarge the comparison panel, two recently published maps, obtained with the IPSC 9 K SNP array, were included: the peach intraspecific ‘NJ Weeping’ x ‘Bounty’ F_2_ map [85] (WxB) and the interspecific DvsS BC_2_ map obtained by backcrossing a hybrid between peach and *P. davidiana* as donor with peach as recurrent [81]. Both maps indicate > 80% coverage for each individual chromosome, globally 93% and 95% of Peach v2.0 length, respectively. The physical distances covered by these two latter maps were recalculated based on the Peach v2.0 assembly. The ANOVA comparison was then applied giving significant differences among the seven maps (*F = 7.17: p = 2.249x10* ^*−5*^). The lowest recombination frequency, displayed by the recurrent parent (1.941 cM/Mb), was not significantly different (see *Post hoc* Tuckey’s pairwise comparison test, Table 6) from that of the two interspecific hybrids (TxE and DvsS, 2.148 and 1.954 cM/Mb, respectively). The PxF F_1_ map, having the highest recombination frequency (3.057 cM/Mb), was significantly different from the two interspecific maps (TxE and DvsS) (*p = 0.0266, p = 0.0013* respectively) as well as from PxF recurrent map (*p = 0.0002*) but not significantly different from the other intraspecific maps (CxA and WxB, 2.564 and 2.553 cM/Mb, respectively). The extreme reduction of recombination rate, observed in the PxF recurrent parent (IF7310828, derived from ‘JH Hale’ x ‘Bonanza’) is comparable to that of the interspecific crosses. ‘JH Hale’ is an old cultivar, one of the founders of the so-called modern Western germplasm, carrying the male sterility allele (*Ps*) while ‘Bonanza’ is a dwarf accession. The IF7310828 recurrent parent is classified as an accession with intermediate tree vigor (semidwarf) [86] belonging to the Western subgroup [20]. No differences in the recombination frequency were observed among the other peach F_1_ individuals (PxF, CxA, WxB). The other parents of the crosses analyzed in this study have different origin: ‘Contender’, ‘Ambra’ and ‘Bounty’ all derive from Western breeding programs while Ferganensis belongs to the Eastern subgroup [20] and NJ Weeping is believed to have Japanese origin [85]. Our results are in agreement with those reported in *Arabidopsis thaliana* [87] where recombination rates, calculated in 17 F_2_ populations derived from 18 accessions, do not correlate with genetic distances between intraspecific parental accessions. An exception in this study is the PxF recurrent parent, which displays a reduction in recombination rate similar to that of the interspecific crosses.

**Table 6:** Tuckey's pairwise comparison test among the different maps

	DvsS	WxB	CxA	PxF recurrent	PxF F_1_	PxF
TxE	0.9335	0.6958	0.9596	0.2531	**0.0266**	0.2337
PxF	**0.0201**	0.9843	0.7918	**0.0006**	0.9592	
PxF F_1_	**0.0013**	0.5816	0.2327	**0.0002**		
PxF recurrent parent	0.8660	**0.0055**	**0.0301**			
CxA	0.4103	0.9959				
WxB	0.1362					

Significant *p*-values (α = 0.05) are reported in bold

## Conclusions

In this paper an improved and refined version of the peach genome assembly based on high quality linkage maps and resequencing data is presented. This new assembly release has been improved in terms of completeness and accuracy, including the increase of mapped and oriented sequences, repositioning of misassembled portions, enhancement of contiguity and correction of base errors. High density (referring to the number of markers used) and high resolution (referring to the number of the seedlings of the mapping progeny) maps are important tools to assist WGS efforts. In fact, even if unanchored WGS assemblies are able to catch the full gene complement, they defect in depicting the whole genome view thus being of little use for comparative genomics. The late high-throughput genotyping technologies such as SNP arrays or genotyping-by-sequencing platforms are essential for developing saturated and high-resolution maps in short times with minimal cost, even in species with a narrow genetic base like peach and other self-pollinating species. Third generation and NGS technologies can be efficiently used in newly or already available genome sequences to obtain a high quality assembly.

## Additional files

Additional file 1: TableS1. SSR markers developed in this study from Peach v1.0. For each primer, the locus name, primer sequences, position in Peach v1.0 and v2.0, repeated motif, polymorphism in TxE (monomorphic, BIN or fully mapped) and location in genic or intergenic regions are reported. **Table S2.** SNP markers developed in this study from the resequencing of the CxA F_1_ parent. For each primer, the locus name, position in Peach v1.0 and v2.0, primer sequences, BIN position and location in genic or intergenic regions are reported. **Table S3.** Illumina short reads libraries. All libraries were sequenced as 2x250s using Illumina sequencing. Reads were filtered on quality, screening for reads that were predominantly simple sequence, and residual phix contamination. **Table S4.** TxE linkage map. For each locus, the genetic position in linkage groups and physical position in Peach v2.0 are reported. Only integrated markers are reported. **Table S5.** Sequenom SNP development and their efficiency with the SNP calling parameters used in this study. *A posteriori* efficiency was calculated using more stringent parameters as in Verde et al. 2013. **Table S6.** CxA linkage map. For each locus, the genetic position in linkage groups and physical position in Peach v2.0 are reported. **Table S7.** PxF linkage map. For each locus, the genetic position in linkage groups and physical position in Peach v2.0 are reported. **Table S8.** Linkage map of PxF F_1_ parent. For each locus, the genetic position in linkage groups and physical position in Peach v2.0 are reported. **Table S9.** Linkage map of PxF recurrent parent (IF7310828). For each locus, the genetic position in linkage groups and physical position in Peach v2.0 are reported. **Table S10.** Composition of the 8 Peach v2.0 pseudomolecules. Start and end of each scaffold, total scaffold length, scaffold denomination in v2.0 and in v1.0, v1.0 position of each v2.0 scaffold, length of the scaffold ends uncovered with markers and mapping and orientation information achieved with the linkage maps used in this study, are reported. **Table S11.** Linkage map of LG6 of MDxSD. For each locus, the genetic position (cM) in linkage groups and physical position in Peach v2.0 are reported. **Table S12.** Peach v2.0 assembly statistics (N/L) prior to the pseudomolecules build. **Table S13.** Peach v2.0 assembly statistics post chromosome-scale build. (XLSX 391 kb)
Additional file 2: Figure S1. Anchoring of the peach scaffolds to the three genetic maps. Colored bars represent the 8 linkage groups: pink for PxF, purple for TxE and blue for CxA. WGS scaffolds were positioned in each pseudomolecule (Pp01 to Pp08) with the corresponding genetic markers and are depicted in three different colors (dark blue, pink and pale blue); genetic markers are in the same colors of the corresponding WGS scaffolds. The zero (0) denotes the six scaffolds placed with random orientation along the pseudomolecules. The asterisk (*) indicates the two scaffolds with random order. The crosshatch (#) indicates the two scaffolds with the wrong order in Peach v2.0 that need to be inverted in a future release. (PDF 495 kb)
Additional file 3: Figure S2. MareyMap plot of PxF linkage maps (including the F_1_ and recurrent parent maps). Vertical bars indicate the putative position of the centromere. The solid line represents the recombination rate plotted along the 8 pseudomolecules calculated using the cubic spline method. (PDF 405 kb)
Additional file 4: Figure S3. MareyMap plot of TxE linkage map. Vertical bars indicate the putative position of the centromere. The solid line represents the recombination rate plotted along the 8 pseudomolecules calculated using the cubic spline method. (PDF 236 kb)
Additional file 5: Figure S4. MareyMap plot of CxA linkage map. Vertical bars indicate the putative position of the centromere. The solid line represents the recombination rate plotted along the 8 pseudomolecules calculated using the cubic spline method. (PDF 233 kb)
