# Supplementary figures and images for: The Peach v2.0 release: high-resolution linkage mapping and deep resequencing improve chromosome-scale assembly and contiguity
Source: BMC Genomics. 2017 Mar 11;18:225. doi: 10.1186/s12864-017-3606-9 (PMC5346207; doi:10.1186/s12864-017-3606-9)

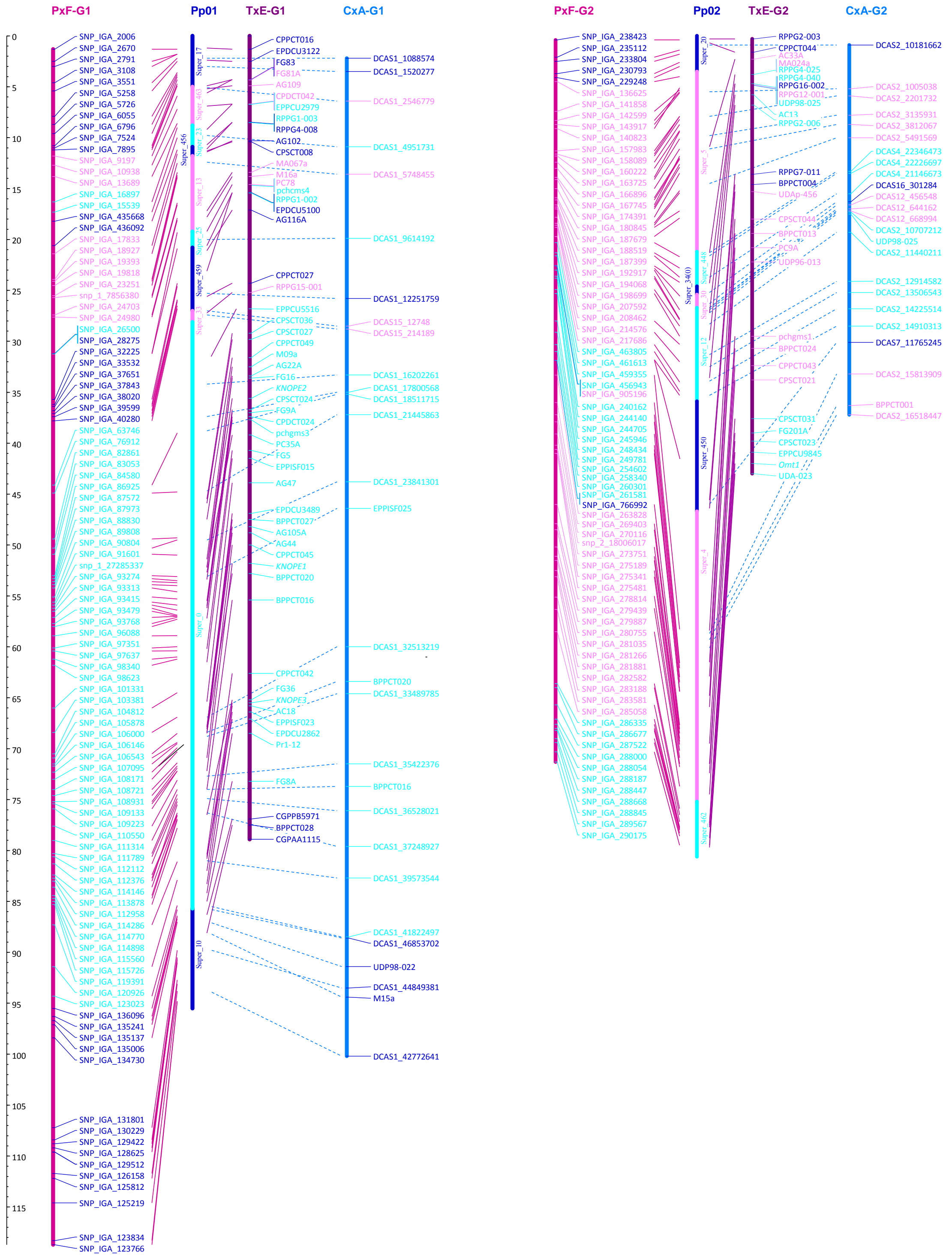

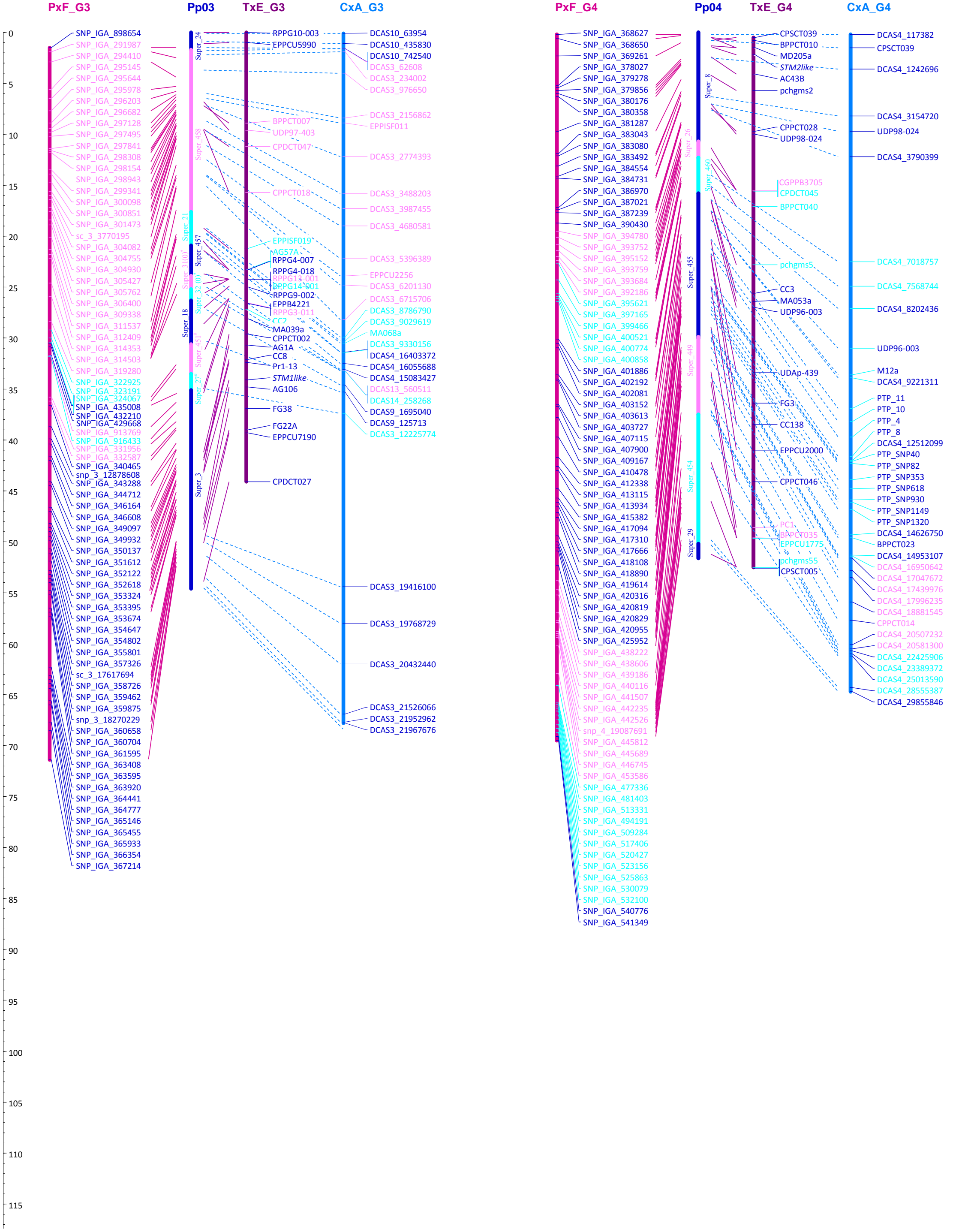

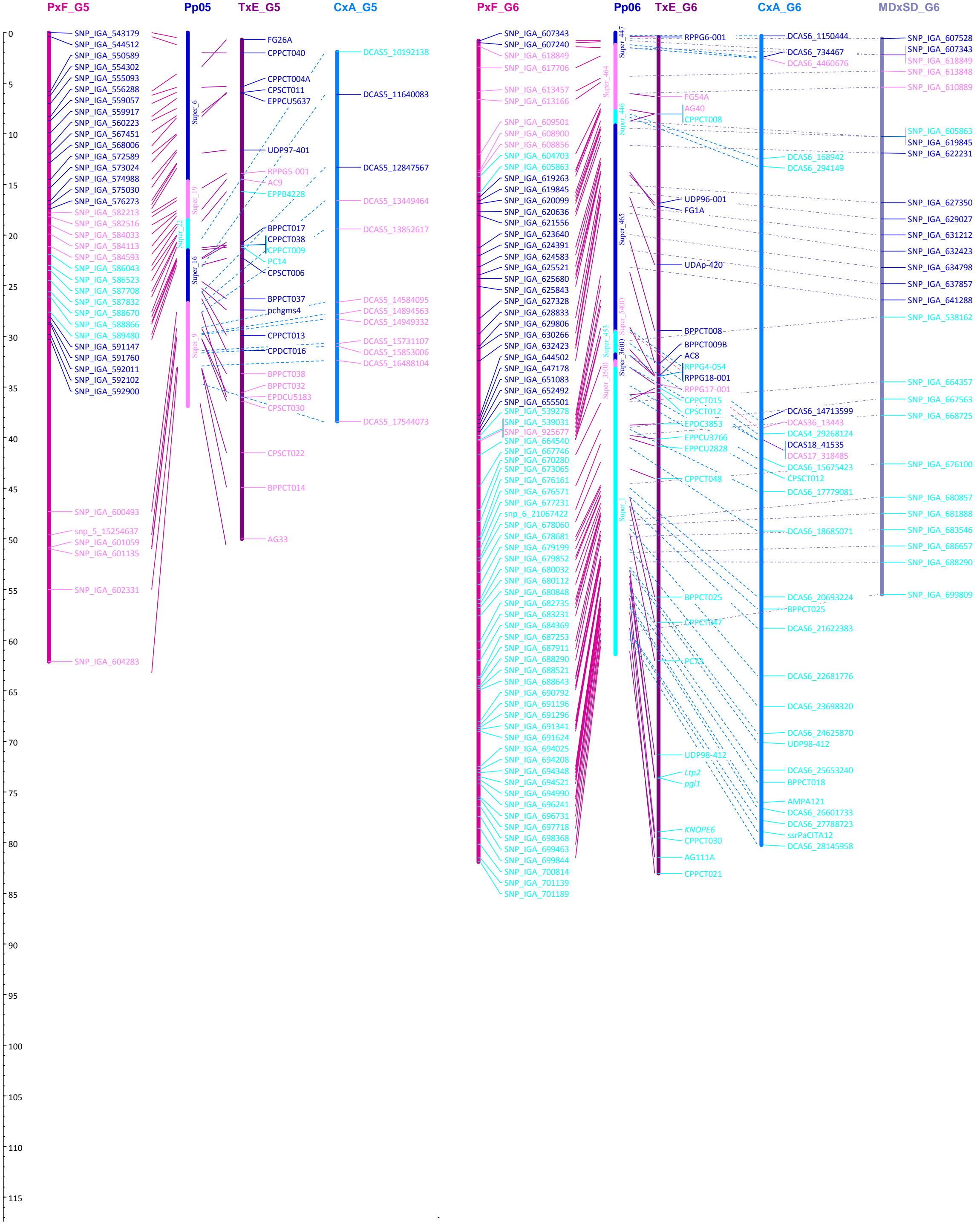

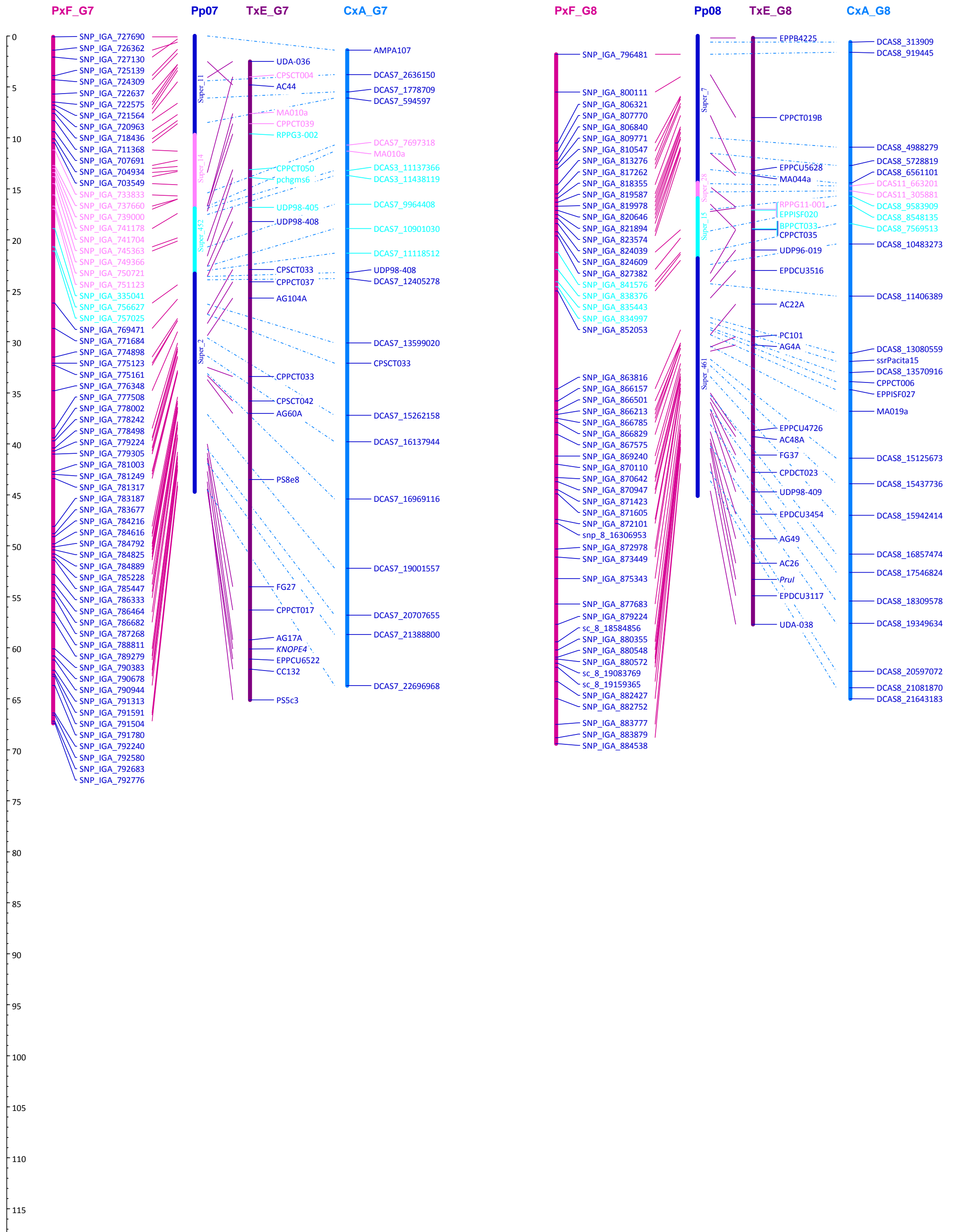

Supplement: Additional file 2: Figure S1. — Anchoring of the peach scaffolds to the three genetic maps. Colored bars represent the 8 linkage groups: pink for PxF, purple for TxE and blue for CxA. WGS scaffolds were positioned in each pseudomolecule (Pp01 to Pp08) with the corresponding genetic markers and are depicted in three different colors (dark blue, pink and pale blue); genetic markers are in the same colors of the corresponding WGS scaffolds. The zero (0) denotes the six scaffolds placed with random orientation along the pseudomolecules. The asterisk (*) indicates the two scaffolds with random order. The crosshatch (#) indicates the two scaffolds with the wrong order in Peach v2.0 that need to be inverted in a future release. (PDF 495 kb) [file 12864_2017_3606_MOESM2_ESM.pdf]

PxF Pp01

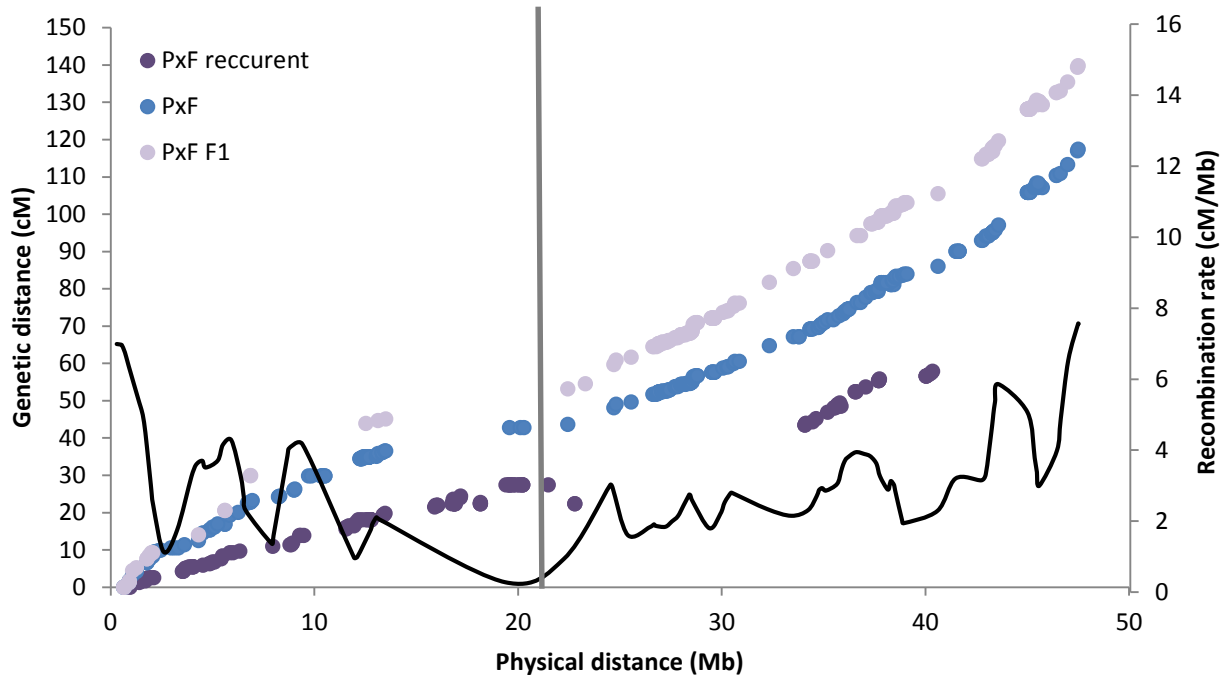

PxF Pp02

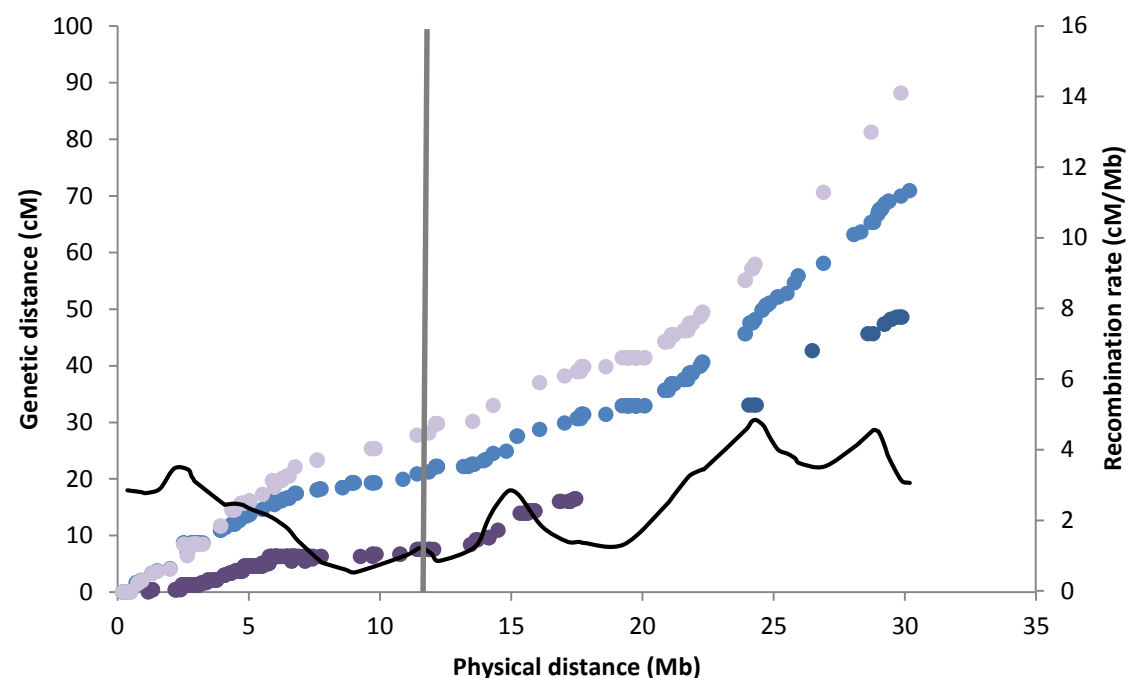

PxF Pp03

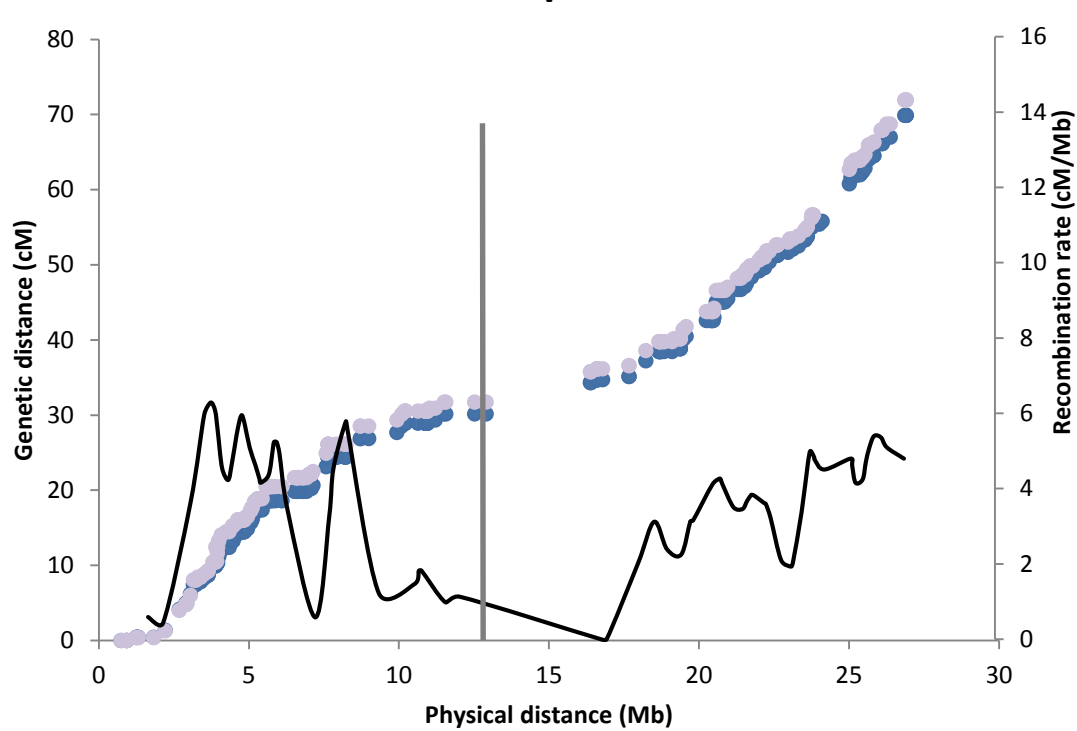

PxF Pp04

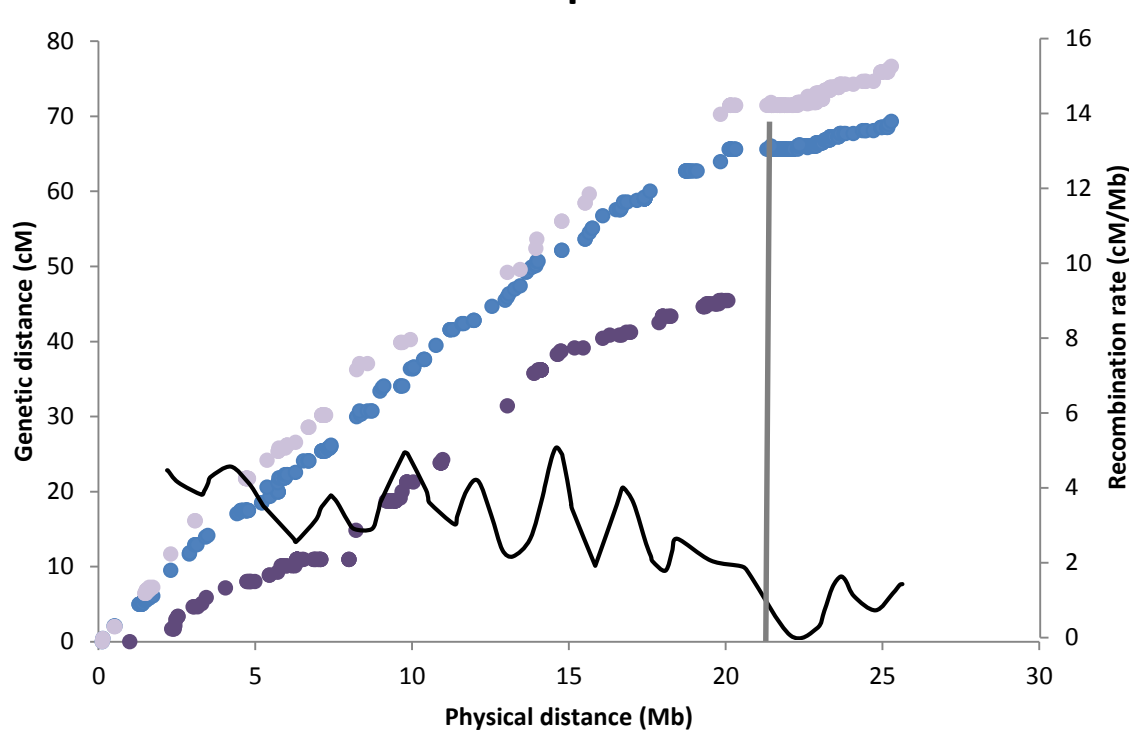

PxF Pp05

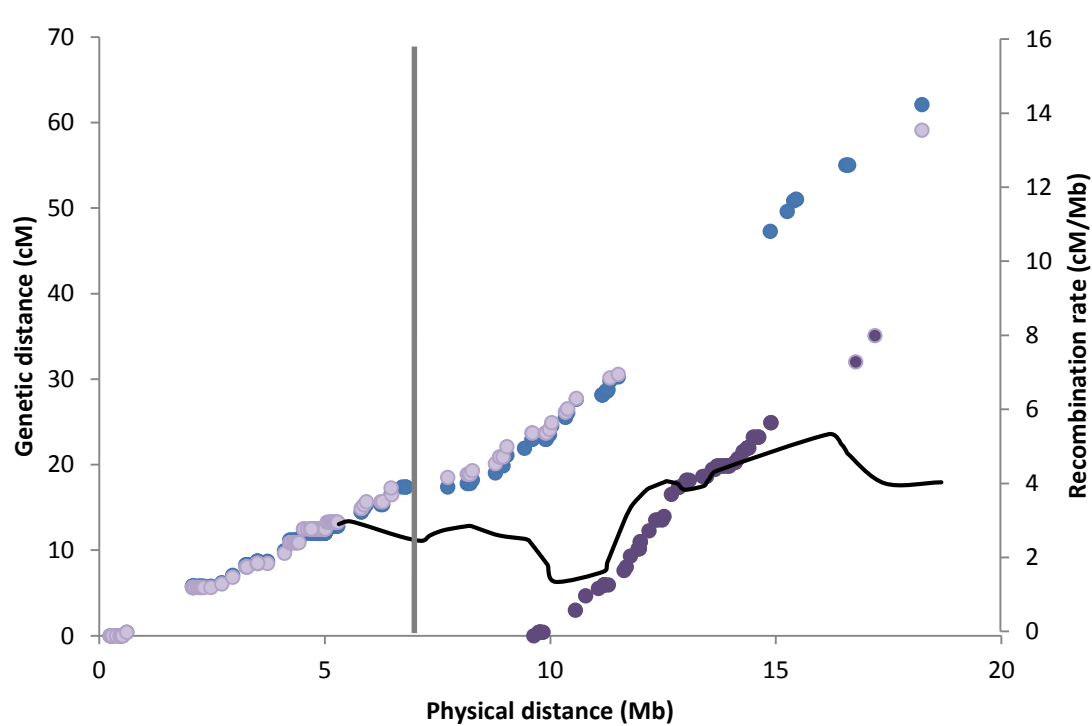

PxF Pp06

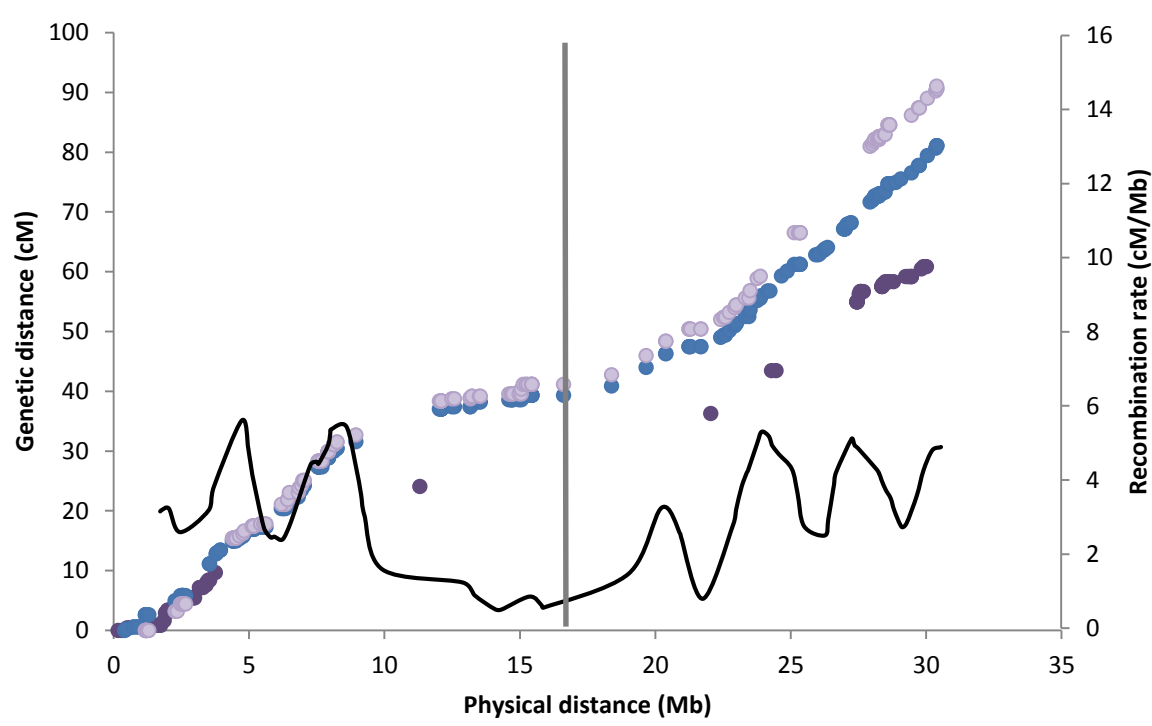

PxF Pp07

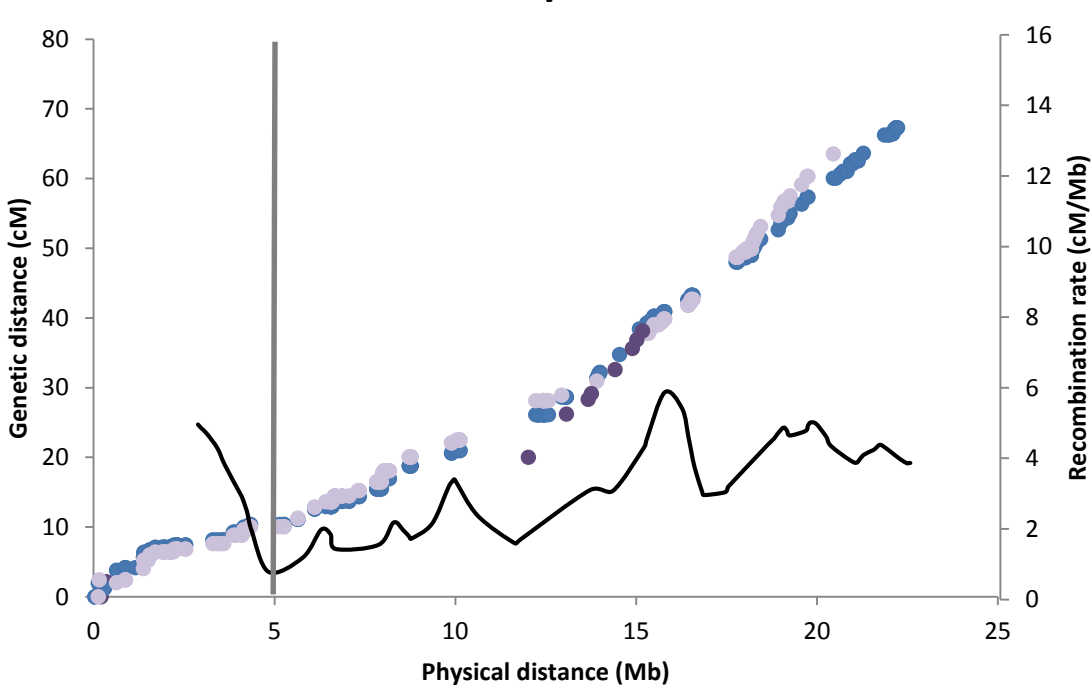

PxF Pp08

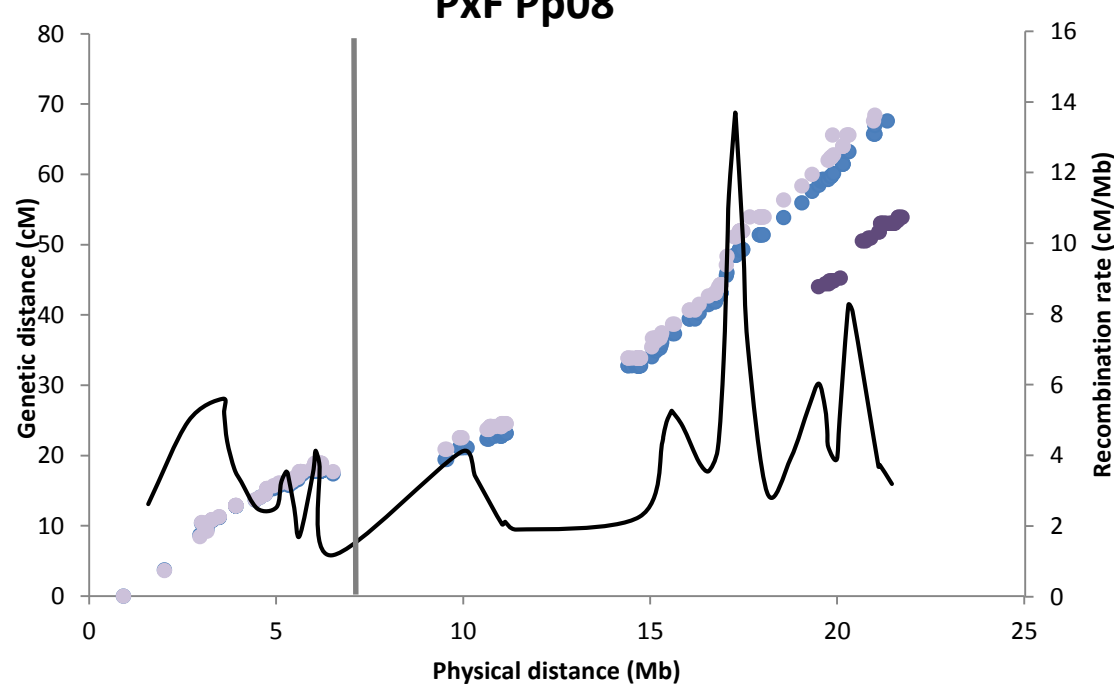

Supplement: Additional file 3: Figure S2. — MareyMap plot of PxF linkage maps (including the F1 and recurrent parent maps). Vertical bars indicate the putative position of the centromere. The solid line represents the recombination rate plotted along the 8 pseudomolecules calculated using the cubic spline method. (PDF 405 kb) [file 12864_2017_3606_MOESM3_ESM.pdf]

TxE Pp01

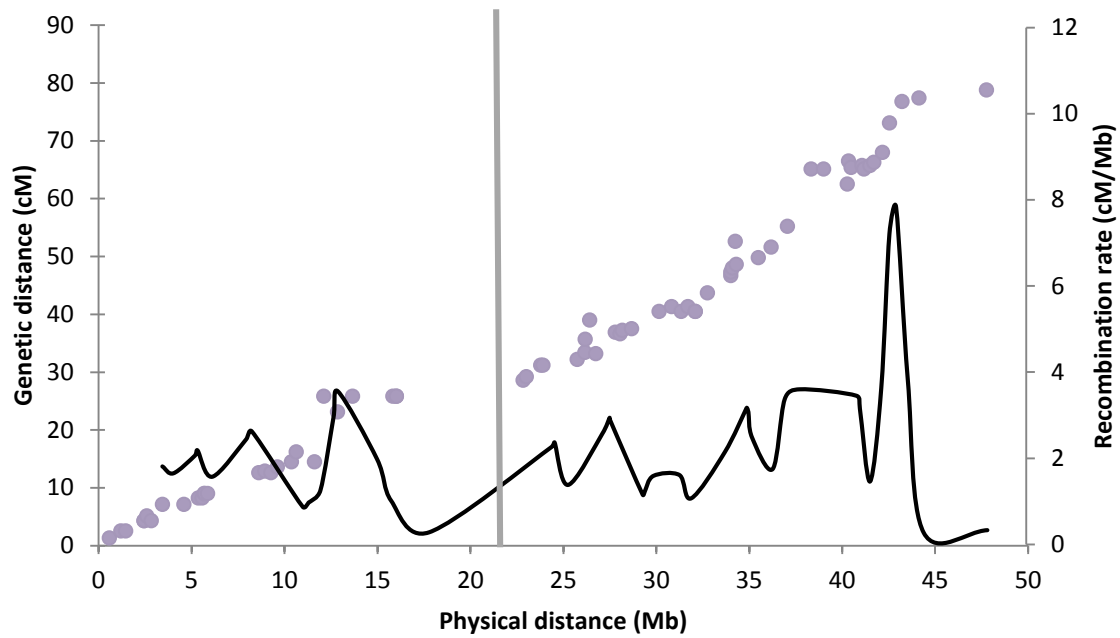

TxE Pp02

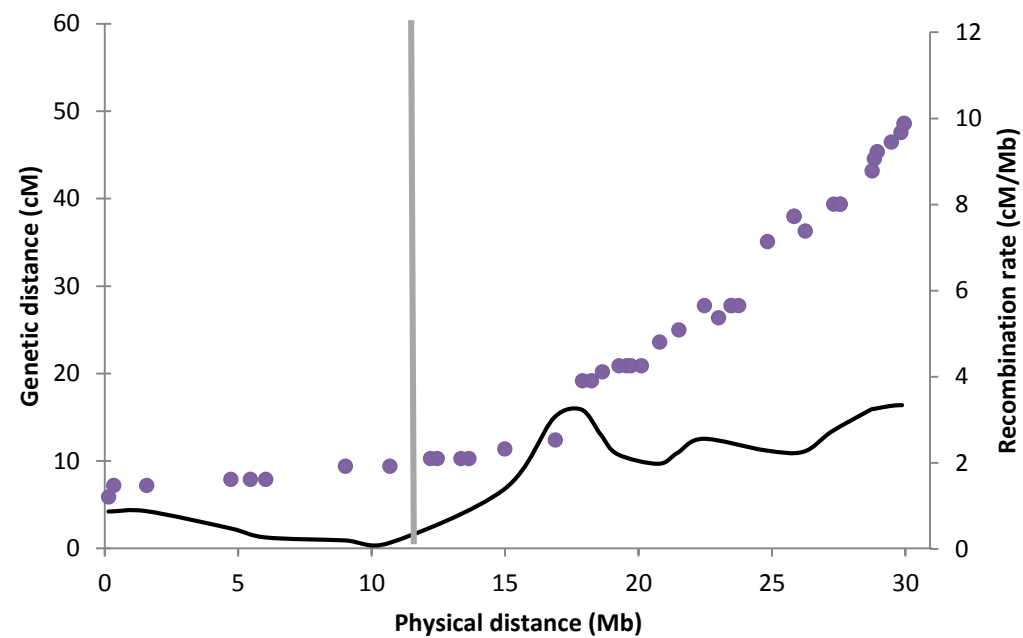

TxE Pp03

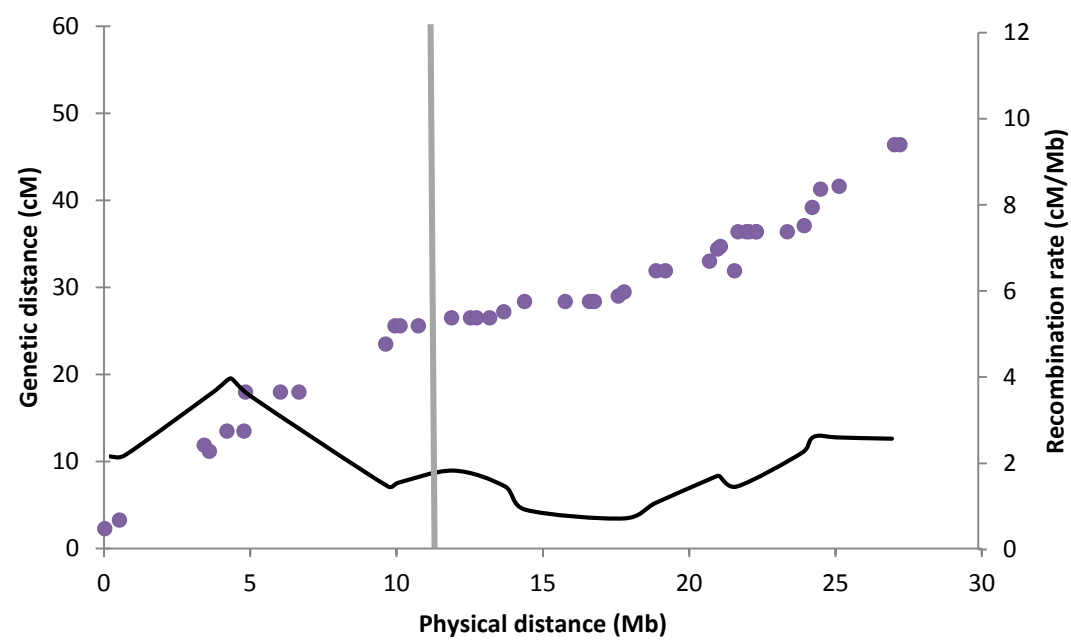

TxE Pp04

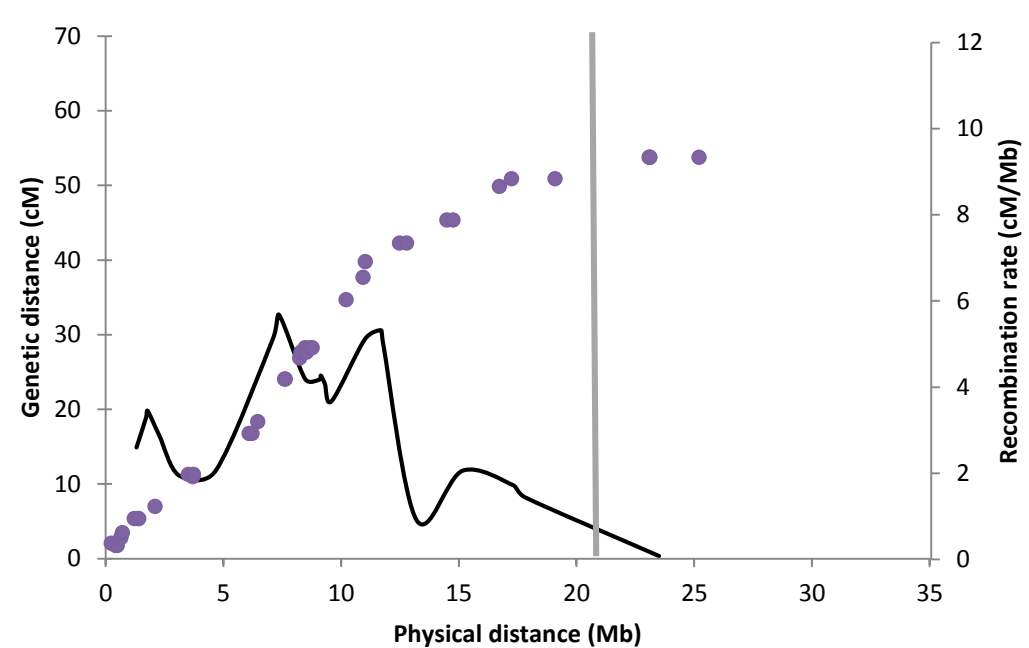

TxE Pp05

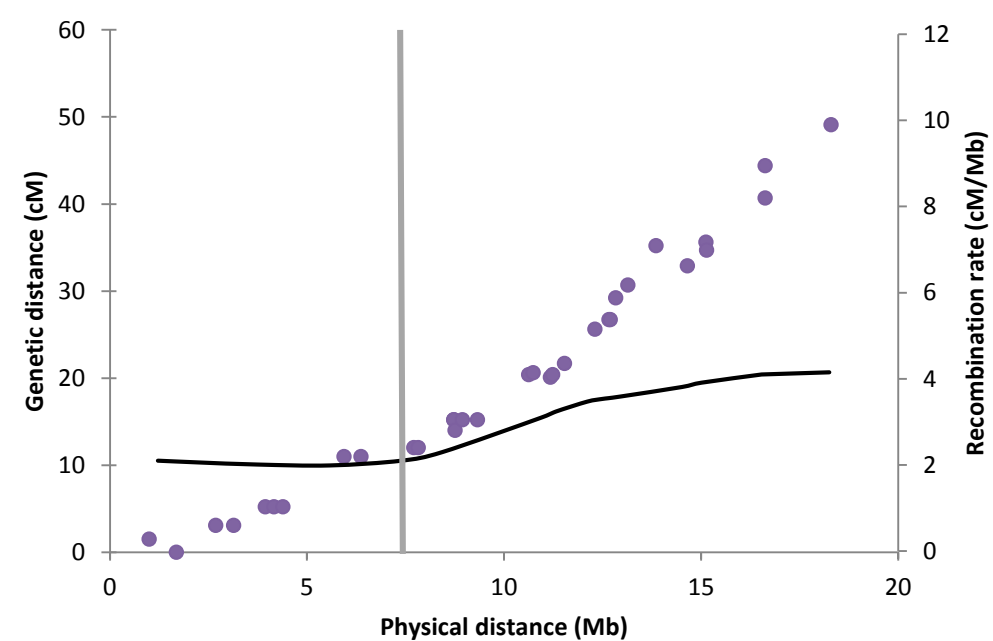

TxE Pp06

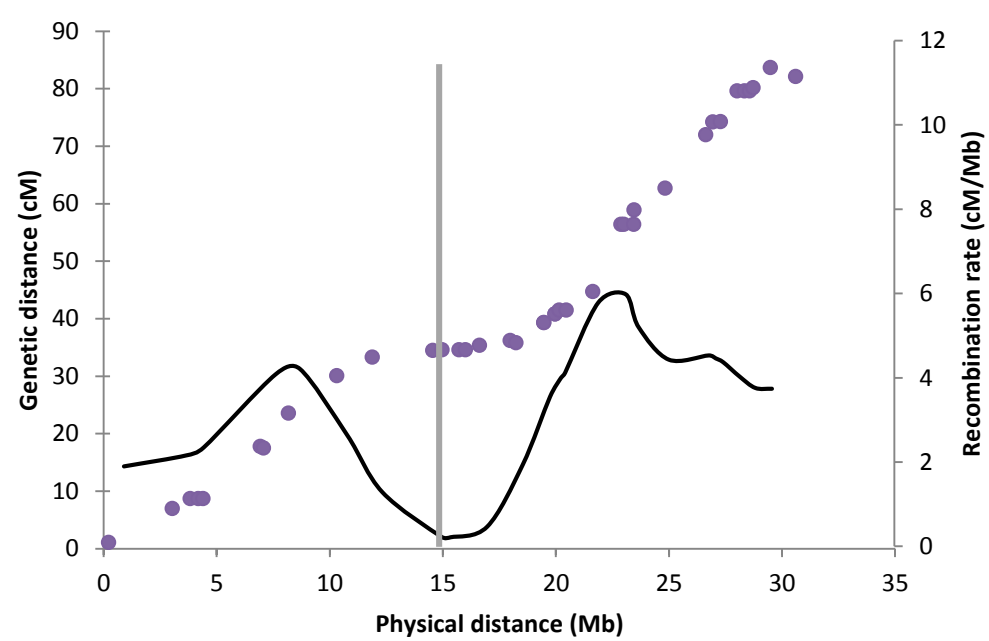

TxE Pp07

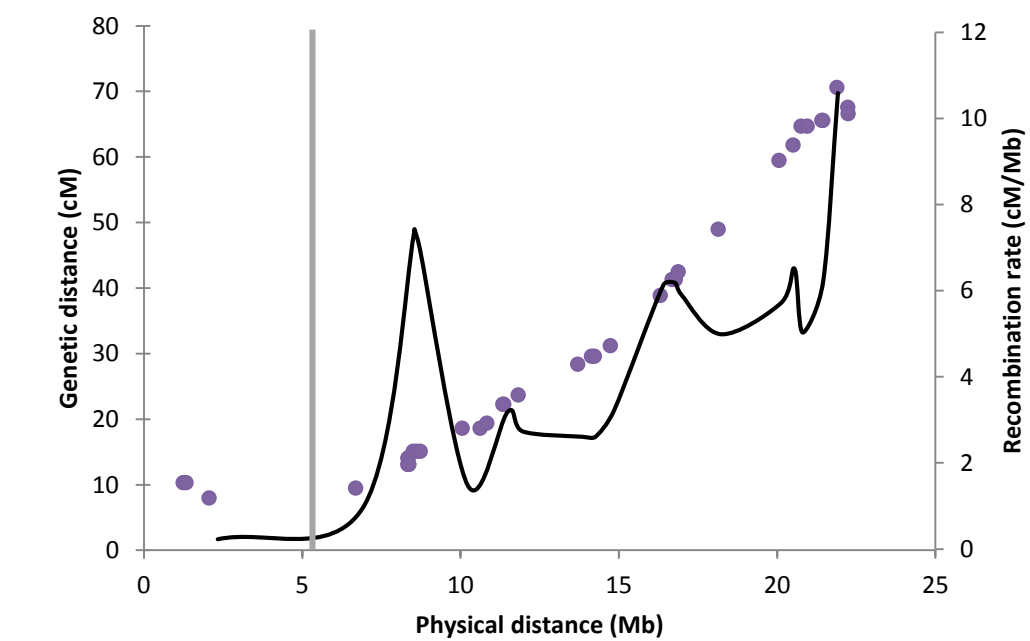

TxE Pp08

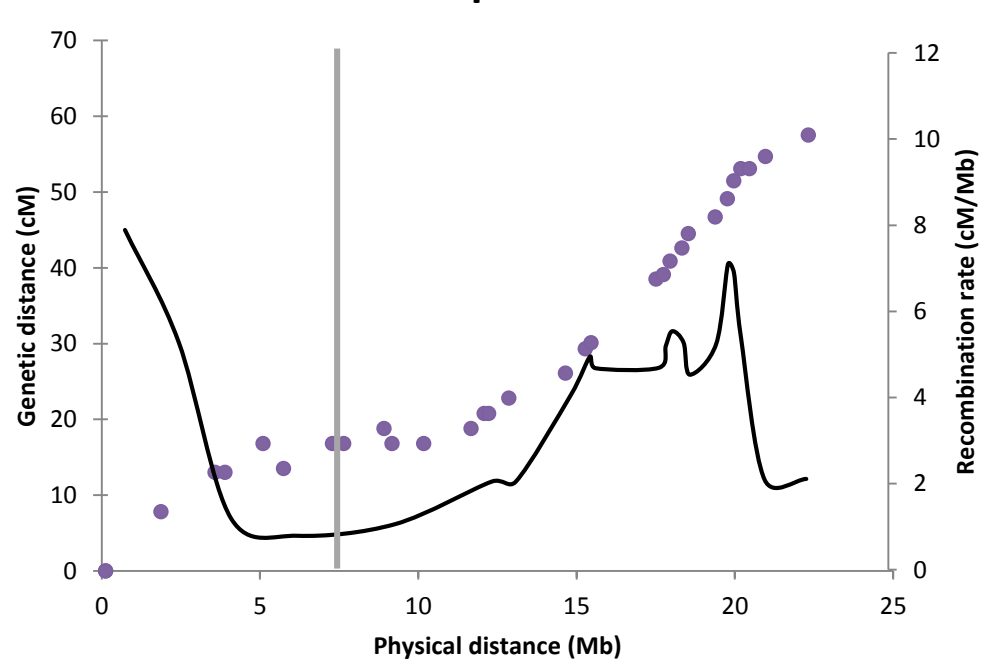

Supplement: Additional file 4: Figure S3. — MareyMap plot of TxE linkage map. Vertical bars indicate the putative position of the centromere. The solid line represents the recombination rate plotted along the 8 pseudomolecules calculated using the cubic spline method. (PDF 236 kb) [file 12864_2017_3606_MOESM4_ESM.pdf]

CxA Pp01

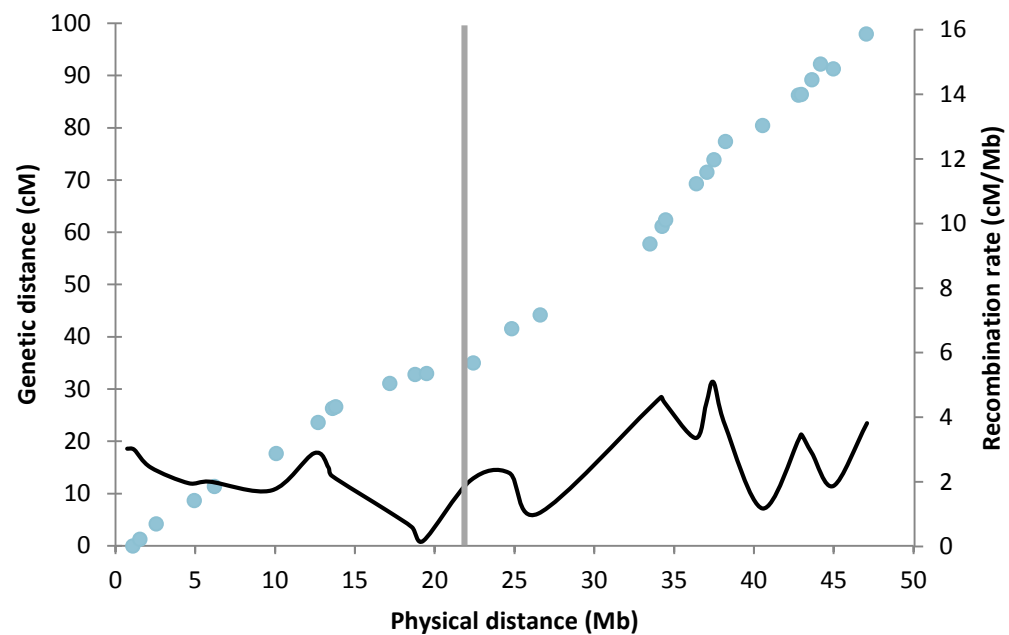

CxA Pp02

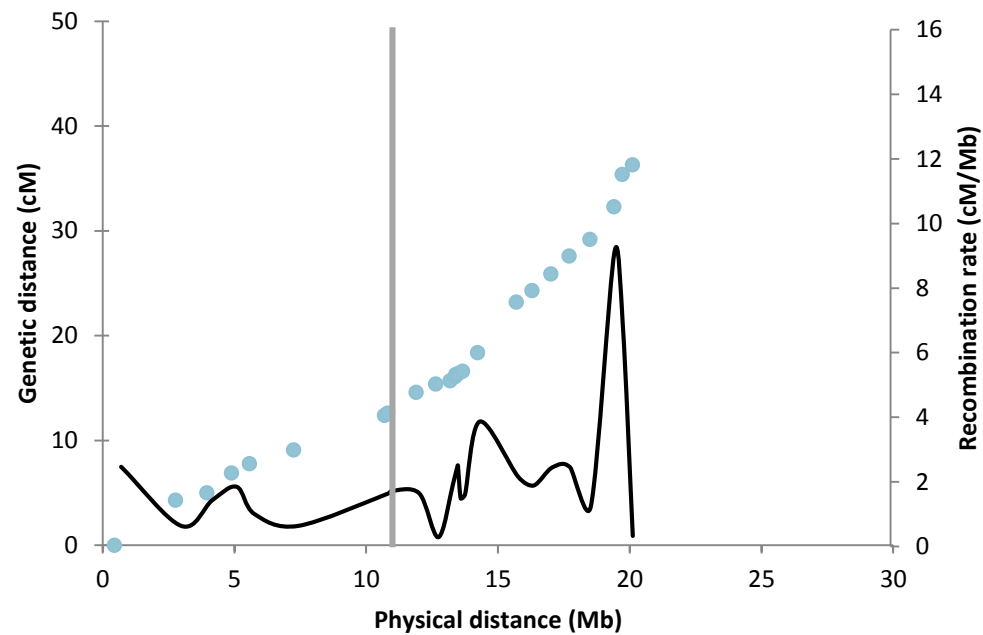

CxA Pp03

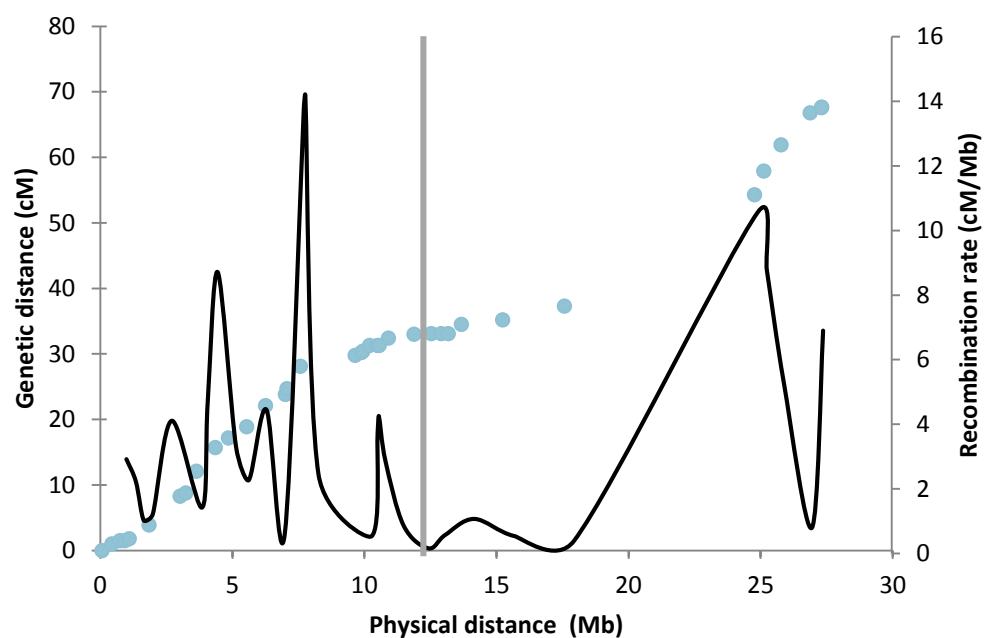

CxA Pp04

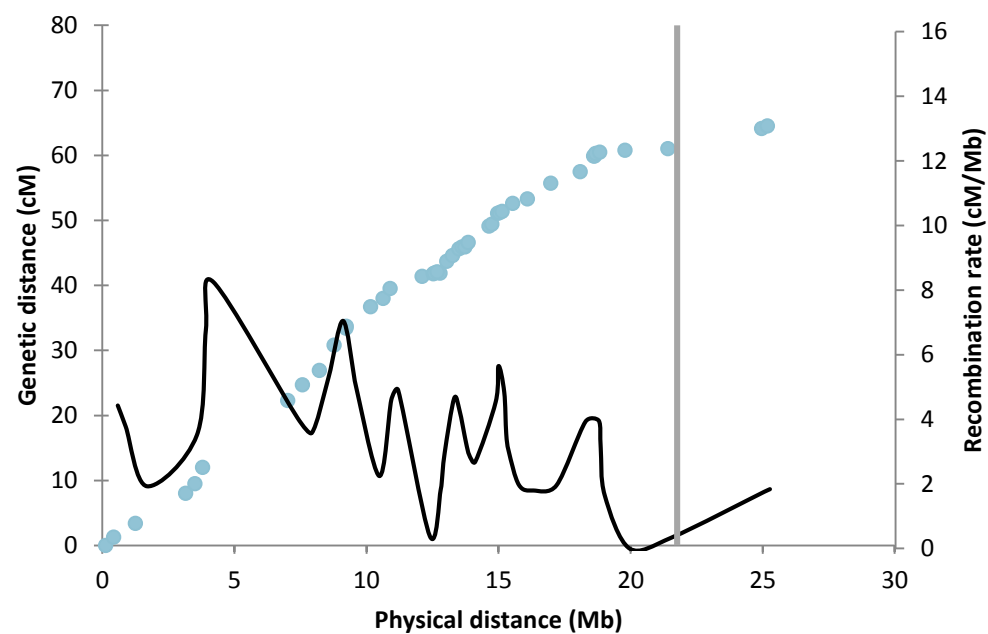

CxA Pp05

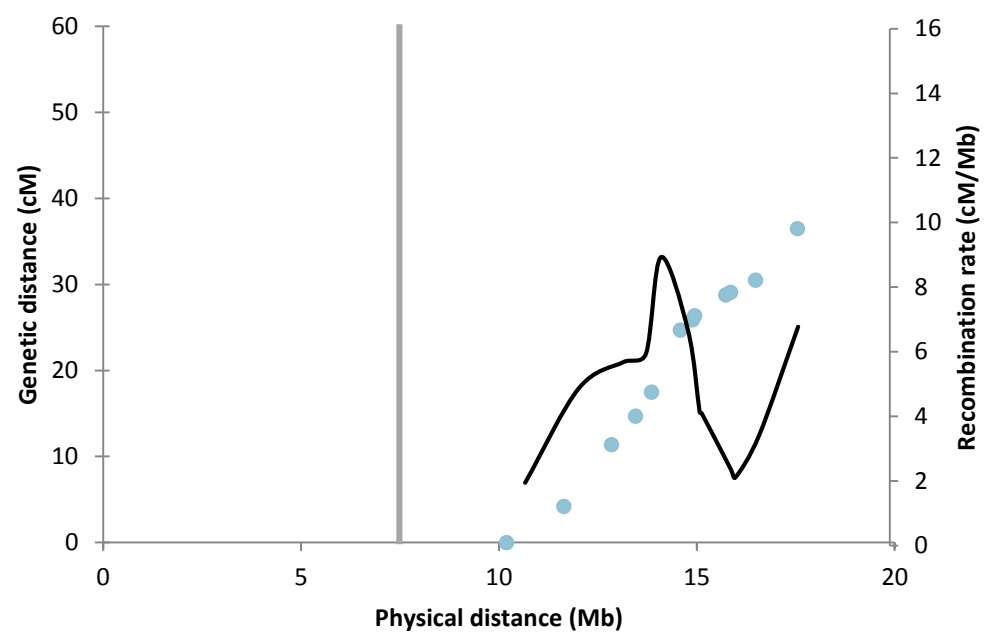

CxA Pp06

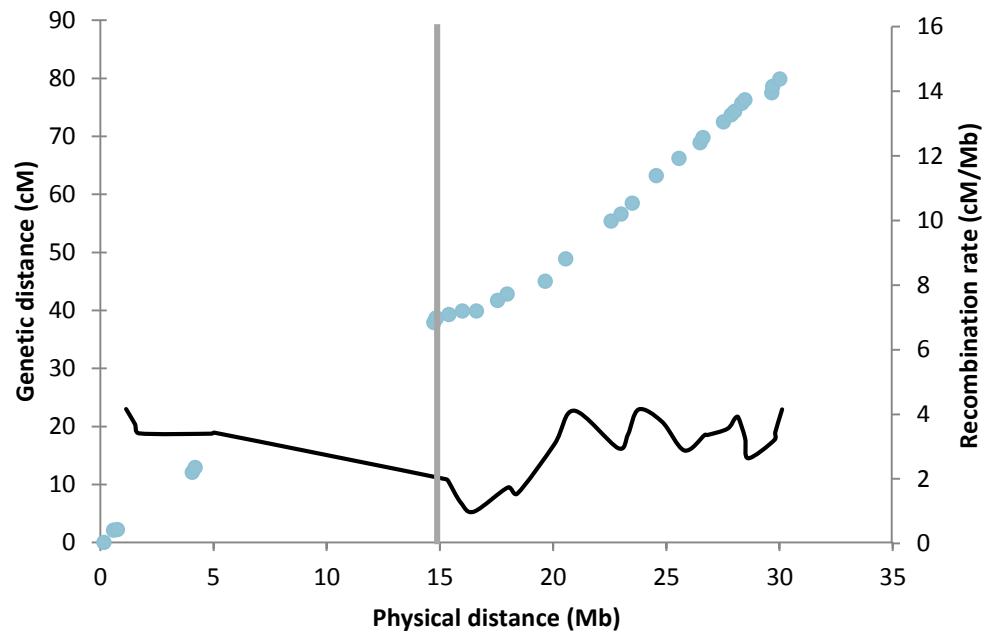

CxA Pp07

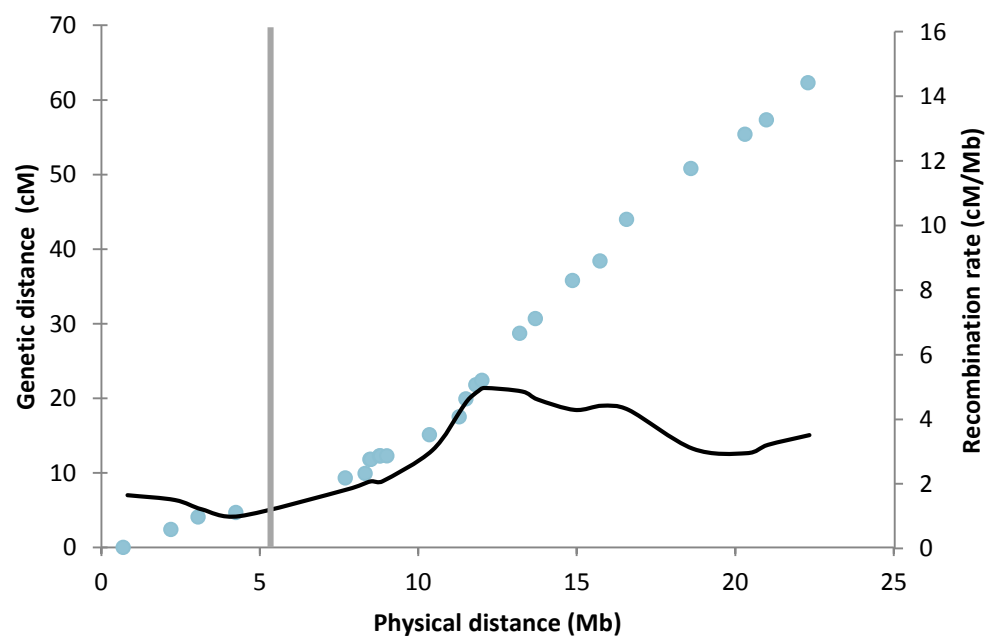

CxA Pp08

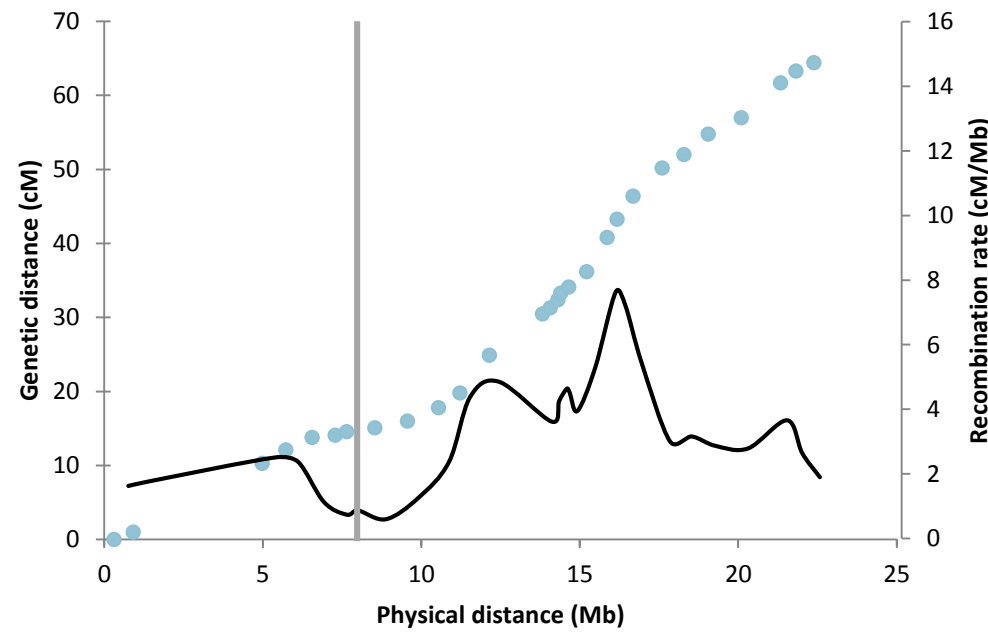

Supplement: Additional file 5: Figure S4. — MareyMap plot of CxA linkage map. Vertical bars indicate the putative position of the centromere. The solid line represents the recombination rate plotted along the 8 pseudomolecules calculated using the cubic spline method. (PDF 233 kb) [file 12864_2017_3606_MOESM5_ESM.pdf]
